# Whole genome sequencing of mouse lines divergently selected for fatness (FLI) and leanness (FHI) revealed several genetic variants as candidates for novel obesity genes

**DOI:** 10.1007/s13258-024-01507-9

**Published:** 2024-03-14

**Authors:** Martin Šimon, Špela Mikec, Santosh S. Atanur, Janez Konc, Nicholas M. Morton, Simon Horvat, Tanja Kunej

**Affiliations:** 1 Chair of Genetics, Animal Biotechnology and Immunology, Department of Animal Science, Biotechnical Faculty, University of Ljubljana, Domžale, 1230 Slovenia; 2 Faculty of Medicine, Department of Metabolism, Digestion and Reproduction, Imperial College London, London, SW7 2AZ UK; 3 Centre for Genomic and Experimental Medicine, University of Edinburgh, Edinburgh, EH4 2XU UK; 4 Laboratory for Molecular Modeling, National Institute of Chemistry, Ljubljana, 1000 Slovenia; 5 The Queen’s Medical Research Institute, Centre for Cardiovascular Science, University of Edinburgh, Edinburgh, EH4 2XU UK

**Keywords:** Obesity, Mouse models, Whole-genome sequencing, Single-nucleotide polymorphism

## Abstract

**Background:**

Analysing genomes of animal model organisms is widely used for understanding the genetic basis of complex traits and diseases, such as obesity, for which only a few mouse models exist, however, without their lean counterparts.

**Objective:**

To analyse genetic differences in the unique mouse models of polygenic obesity (Fat line) and leanness (Lean line) originating from the same base population and established by divergent selection over more than 60 generations.

**Methods:**

Genetic variability was analysed using WGS. Variants were identified with GATK and annotated with Ensembl VEP. g.Profiler, WebGestalt, and KEGG were used for GO and pathway enrichment analysis. miRNA seed regions were obtained with miRPathDB 2.0, LncRRIsearch was used to predict targets of identified lncRNAs, and genes influencing adipose tissue amount were searched using the IMPC database.

**Results:**

WGS analysis revealed 6.3 million SNPs, 1.3 million were new. Thousands of potentially impactful SNPs were identified, including within 24 genes related to adipose tissue amount. SNP density was highest in pseudogenes and regulatory RNAs. The Lean line carries SNP rs248726381 in the seed region of *mmu-miR-3086-3p*, which may affect fatty acid metabolism. KEGG analysis showed deleterious missense variants in immune response and diabetes genes, with food perception pathways being most enriched. Gene prioritisation considering SNP GERP scores, variant consequences, and allele comparison with other mouse lines identified seven novel obesity candidate genes: *4930441H08Rik*, *Aff3*, *Fam237b*, *Gm36633*, *Pced1a*, *Tecrl*, and *Zfp536*.

**Conclusion:**

WGS revealed many genetic differences between the lines that accumulated over the selection period, including variants with potential negative impacts on gene function. Given the increasing availability of mouse strains and genetic polymorphism catalogues, the study is a valuable resource for researchers to study obesity.

**Supplementary Information:**

The online version contains supplementary material available at 10.1007/s13258-024-01507-9.

## Introduction

Genome sequencing has become an important approach for exploring gene functions, phenotypical diversity among individuals and populations, and personalised medicine (Lu et al. 2014; Uffelmann et al. 2021). Genome-wide association studies (GWAS) have identified thousands of genetic variants associated with the increased risk for various human diseases (Sun et al. 2022).

Obesity, considered by many a 21st-century epidemic, has been widely believed to result from the disequilibrium between energy intake and expenditure. However, the aetiology of obesity is more complex, resulting from various factors, including genetic predispositions (González-Muniesa et al. 2017). It significantly increases the risk of various diseases such as type II diabetes mellitus, fatty liver disease, hypertension, myocardial infarction, stroke, dementia, osteoarthritis, and several cancers, thereby declining one’s life quality and life expectancy (Blüher 2019), as well as increasing the economic burden of the country (Pineda et al. 2018).

Studies have estimated that between 40% and 70% of obesity is heritable. Obesity is broadly divided into two categories: the monogenic type, which results from chromosomal deletions or single gene defects with a large effect, and the more common polygenic type, in which hundreds of genetic polymorphisms, each having a small effect, contribute to the obesity phenotype. The latter type follows a similar pattern of heritability as other complex traits and diseases (Loos and Yeo 2022).

Analysing genomes of animal model organisms, such as the mouse (*Mus musculus*), has been widely used to improve animal production and to understand the genetic basis of complex traits and diseases in both animals and humans (Andersson and Georges 2004; Saul et al. 2019). There are few mouse models for monogenic (for example *ob*/*ob*, *db*/*db*, and *fa*/*fa*), as well as polygenic types of obesity (for example NZO, TSOD, and C3H) (Suleiman et al. 2020). However, the latter strains, have no lean counterparts derived from the same base population. Prolonged selective breeding of inbred mice for desired divergent phenotypes produces novel, polygenic, and reproducible models of disease (Saul et al. 2019; Palma-Vera et al. 2022).

In this study, we present the results of whole-genome sequencing (WGS) analysis focused on single nucleotide polymorphisms (SNPs) in two unique mouse models for polygenic obesity (FLI, Fat line) and leanness (FHI, Lean line) which were established from the same base population using divergent selection on body fat percentage for over 60 generations (Sharp et al. 1984), differing 5-6-fold in fat content at the end of the selection process (Bünger and Hill 1999). Horvat et al. (2000) identified four major quantitative trait loci (QTL) responsible for their divergent phenotype, which is independent of known mutations in the leptin system or individual obesity-associated gene loci such as *Cpe* and *Ay*. Subsequent studies led to the identification of the *Deptor* gene as a novel regulator of adipogenesis (Laplante et al. 2012) and mitochondrial thiosulfate sulfurtransferase (*Tst*) as the causative gene for resistance to obesity (Morton et al. 2016). The identified polymorphisms and their corresponding genes identified in this study provide novel candidates and represent a valuable resource for researchers to study obesity.

## Materials and methods

### Mouse selection lines

Starting with a base population of two inbred mouse lines (JU, CBA) and one outbred mouse line (CFLP), two unique mouse models were developed following more than 60 generations of divergent selection for increased (FLI, Fat line) or decreased (FHI, Lean line) body fat percentage (Sharp et al. 1984). At the end of the selection process, the initial value of approximately 10% body fat in generation 0 increased to 22% in the Fat line and decreased to 4% in the Lean line, differing by a factor of 5–6. In the 53^rd^ generation, the divergence between lines was 18% or a factor of 5–6, corresponding to about six standard deviations. This divergence in body fat percentage was shown to be due to the gradual accumulation of “obese” alleles in the Fat line and “lean” alleles in the Lean line (Bünger and Hill 1999). Subsequently, inbred lines were established from both lines by full-sib mating. Mice were maintained at The Centre for Laboratory Animals at the University of Ljubljana, Slovenia, the only research facility maintaining the Fat and Lean mouse lines. Animals were housed in individually ventilated cages (IVC), with tunnels for handling and nesting material as enrichment. They received a regular chow diet and had unlimited access to water under controlled environmental conditions: temperature (21 °C ± 2 °C), humidity (40–70%), and light (12 h day:12 h night cycle).

### Whole-genome sequencing to explore genomic variability

Mouse strains are considered inbred after being maintained by successive brother-to-sister matings for at least 20 generations when on average at least 98.6% of the loci in each mouse are homozygous (Beck et al. 2000). In the present study whole genome sequencing (WGS) analysis was performed on spleen DNA samples from the Fat and Lean mouse lines in the 70th and 68th generation of inbreeding, respectively, as described by Mikec et al. (2022). In short, WGS was performed using the Illumina NextSeq 500 platform at the Medical Council Clinical Sciences Centre, University of Edinburgh, Scotland, UK. Reads were preprocessed and then mapped to the mouse reference genome (version GRCm38.86) using the Burrows-Wheeler Aligner (BWA) alignment tool (Li and Durbin 2009). The Genome Analysis Toolkit (GATK) (McKenna et al. 2010) Best Practices recommendations (Depristo et al. 2011; Van der Auwera et al. 2013) were followed for variants calling. Called SNPs were filtered to obtain SNPs with variant confidence standardised by depth above 2 (QD < 2.0; QualByDepth), quality above 30 (QUAL < 30), median mapping quality of reads supporting that site above 40 (MQ < 40.0; RMSMappingQuality), strand odds ratio under 3 (SOR > 3.0; StrandOddsRatio), Fisher’s exact test (FS) for strand bias under 60 (FS > 60.0), rank sum test for mapping qualities of reference vs. alternative reads above − 12.5 (MQRankSum < -12.5), rank sum test for site position within reads above − 8.0 (ReadPosRankSum < -8.0), and coverage depth above 30. Indels were filtered using QD < 2.0, QUAL < 30.0, FS > 200.0, ReadPosRankSum < -20.0, and SOR > 10.0. Finally, the identified variants were annotated using the Ensembl Variant Effect Predictor (VEP) (https://www.ensembl.org/Tools/VEP) (McLaren et al. 2016). Impact of SNPs are according to SnpEff (Cingolani et al. 2012b) and SnpSift (Cingolani et al. 2012a). Only homozygous polymorphisms were included in the analysis. Selected variants were validated by Sanger sequencing. The primer pairs used for PCR are given in Mikec et al. (2022), Šimon et al. (2024) and Supplementary Table S1. Sequence alignment was done using MEGA11: Molecular Evolutionary Genetics Analysis version 11 (Tamura, Stecher, and Kumar 2021).

### Bioinformatics analyses

For bioinformatics analyses, we primarelly focused on line-specific SNPs (alleles present in one line but not in the other, compared to the mouse reference genome). The g.Profiler (Raudvere et al. 2019), WebGestalt (Liao et al. 2019), and Kyoto Encyclopedia of Genes and Genomes - KEGG (Kanehisa and Goto 2000) were used for Gene Ontology and pathway enrichment analysis. Cytoscape (Shannon 2003) and Cytoscape plugin ClueGO (Bindea et al. 2009) were used to visualize KEGG pathways and gene biological functions, respectively. The miRPathDB 2.0 (Kehl et al. 2020) was used to obtain the miRNA seed region, which was visualised using the Golden Helix GenomeBrowse® v3.1.0 visualisation tool (http://www.goldenhelix.com) (Golden Helix, Inc, Bozeman, MT), and the miRTargetLink 2.0 (Kern et al. 2021) was used to retrieve validated targets of miRNAs. The LncRRIsearch tool was used to predict targets of identified lncRNAs (Fukunaga et al. 2019). Genes related to obesity were obtained from the International Mouse Phenotyping Consortium - IMPC database (Birling et al. 2021) using the search keyword “abnormal adipose tissue amount”. QTL intervals were obtained from our previous study (Horvat et al. 2000). Annotations for obesity candidate genes were done for SNPs with Genomic Evolutionary Rate Profiling (GERP) score above six located in regulatory regions (promoters, promoter flanking regions, enhancers, CTCF binding sites, transcription factor binding sites, open chromatin regions) or having a potential moderate (inframe insertion, inframe deletion, missense variant, protein altering variant) or high (splice acceptor variant, splice donor variant, stop gained, frameshift variant, stop lost, start lost, transcript amplification, feature elongation, feature truncation) impact (McLaren et al. 2016), knockout effect from IMPC database, and their biological function, effect on phenotype, involvement of orthologous genes in human diseases related to obesity and its comorbidities using the Mouse Genome Database (MGD) (Bult et al. 2019), and differential expression. The expression data are according to our unpublished microarray analysis done on various tissues and RNA-seq on the hypothalamus. Finally, the potential involvement of SNPs within candidate genes in obesity was validated by comparing alleles between our mice lines and obesity-prone NZO/HlLtJ and obesity-resistant A/J strain.

## Results

**Fig. 1:**
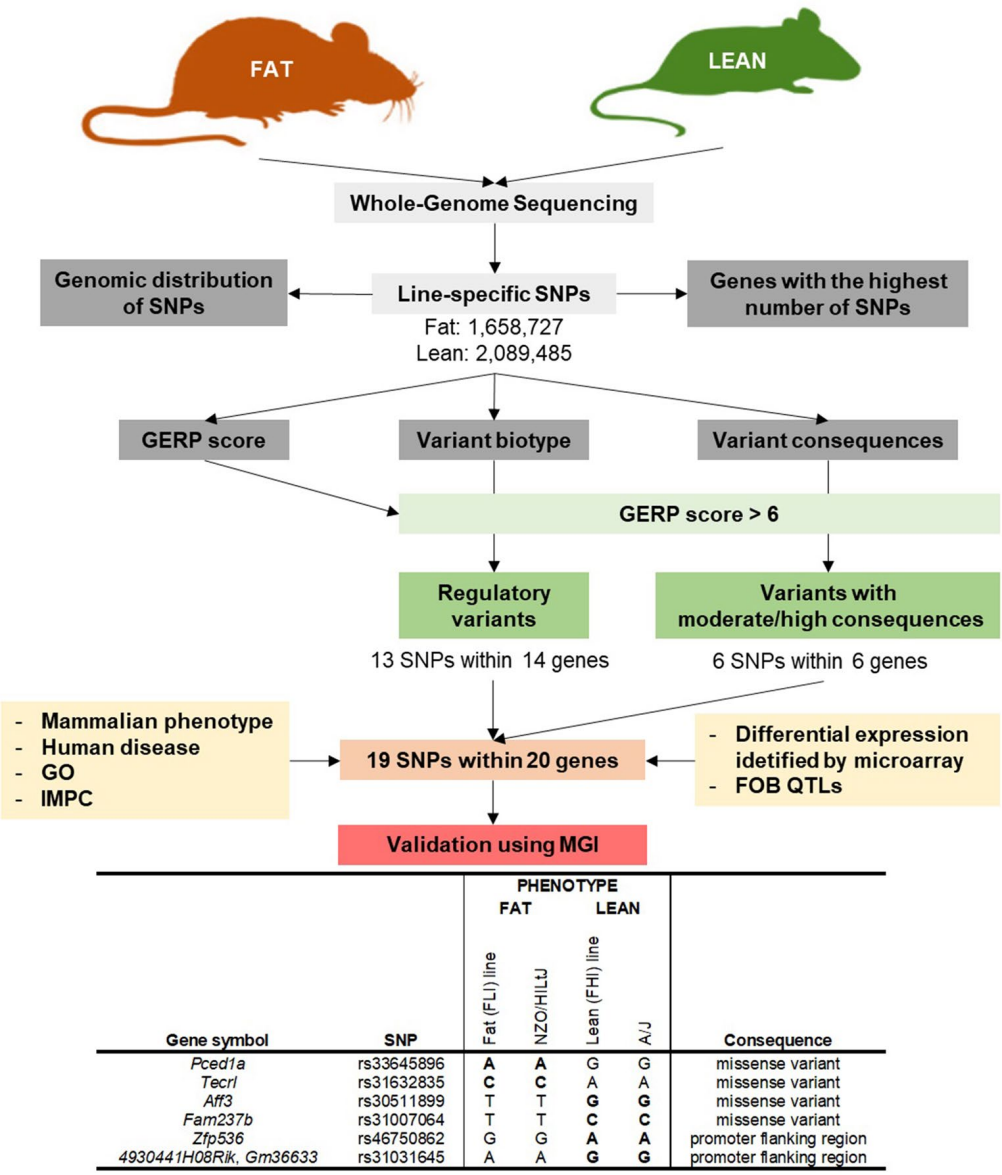
Workflow and main results from whole-genome sequencing of the Fat and Lean mice lines

In the present study, we analysed genomes of Fat and Lean mouse lines, divergently selected for body fat percentage, focusing on line-specific SNPs. Firstly, their genomic distribution was analysed, followed by the identification of genes with the highest number and density of SNPs, location of constrained elements (GERP score), and analysis of variant biotypes and consequences. Then, we focused on regulatory variants and variants with predicted moderate or high impacts and GERP score above 6, resulting in 19 SNPs within 20 genes. For these genes, their previous annotations related to obesity were obtained from a public database, and their positions within previously identified obesity QTLs in our mouse models and their expressions in different tissues were examined. Additionally, the potential importance of identified SNP was validated by comparing the presence of alleles in obesity-resistant A/J and obesity-prone NZO/HlLtJ mice lines. The workflow and main results are shown in Fig. 1.

**Fig. 2:**
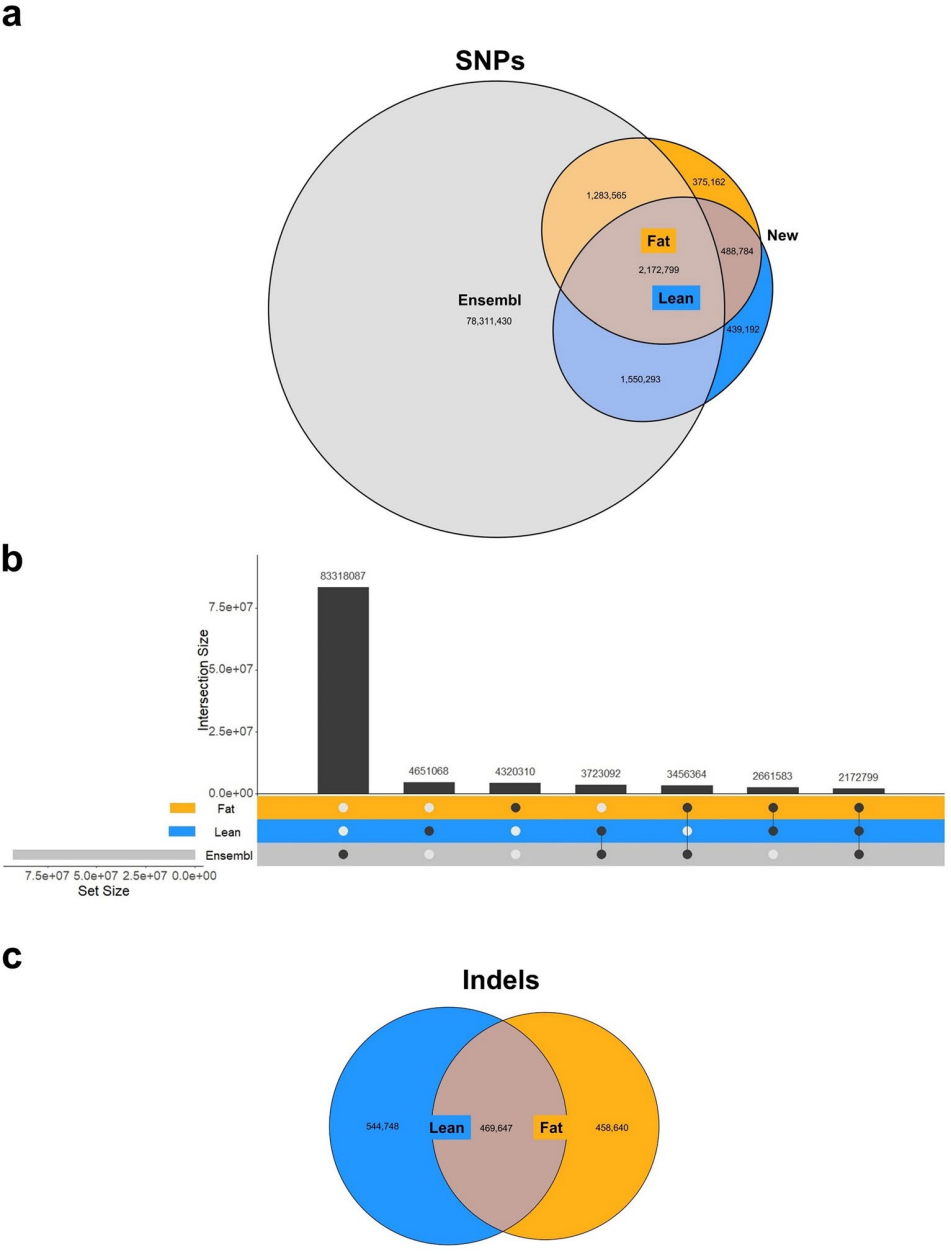
Sequence variants (SNPs and indels) identified in the Lean and Fat mouse selection lines. (**a**) Numbers of private and shared SNPs from the Fat and Lean mouse lines and the Ensembl database. The shape sizes are not proportional to the number of SNPs. (**b**) In scale comparison between SNPs deposited in the Ensembl and identified in the Lean and Fat lines, (**c**) number of indels identified in the Lean and Fat lines

Sequencing genomes of male Lean (FHI line) and Fat (FLI line) mice revealed 4,651,068 and 4,320,310 SNPs in each line respectively among which the majority (2,661,583) are shared between both lines (Fig. 2). Out of 6,309,795 SNPs in total, we identified 1,303,138 SNPs that have not been previously reported (without rs ID). Of those variants, 488,784 are shared by both lines, while 439,192 and 375,162 SNPs are private to the Lean and Fat line, respectively (Fig. 2a, c). Moreover, 1,014,395 and 928,287 insertions and deletions (indels) were identified in the Lean and Fat line, respectively, and 469,647 indels occurred in both (Fig. 2b).

Three selected SNPs were validated using Sanger sequencing and are summarized along with SNPs from Mikec et al. (2022) and Šimon et al. (2024) on Supplementary Fig. S1-3. Among these SNPs, it is worth mentioning a SNP rs37739792 within the intron of *Hif1a* and also overlapping protein-coding gene *Gm15283* (Supplementary Fig. S2).

Out of 1,303,138 novel SNPs, 439,192 and 375,162 were private either for the Lean or the Fat line, respectively, while 488,784 were present in both lines. In total, 180 novel variants with the predicted high-impact and 779 deleterious (including low confidence) missense variants were identified (Supplementary Table S2).

From 1,303,138 novel SNPs, 552,539 (Fat: 164,055, Lean: 178,498, Both: 209,986) are located within 20,600 genes involved in biological processes such as localization, response to stimuli, and signalling/signal transduction (Supplementary Fig. S4a). Meanwhile, the predicted 180 high-impact and 779 deleterious missense variants (DMVs) are located within 676 genes primarily involved in immune response (Supplementary Fig. 4b).

### Distribution of line-specific SNPs

In the Lean line, most line-specific SNPs were located on chromosome 2 (184,531), followed by chromosomes 7 (162,635) and 15 (154,786). In contrast, in the Fat line, chromosome 6 (154,366) has the most SNPs, followed by chromosomes 1 (140,760) and 13 (136,462) (Supplementary Fig. S5a, b). A total of 36 line-specific and novel SNPs (Fat: 15, Lean: 21) were also identified on chromosome Y, of which one and nine were in the introns of genes *Mid1-ps1* and *Gm47283* in the Lean line, respectively (Supplementary Fig. S5c).

### Genes with the most line-specific SNPs

Sixty-four genes (Lean: 25, Fat: 23) contain at least 3000 SNPs, all within protein coding genes. Two genes with more than 3000 line-specific SNPs were identified in both lines, *Macrod2* and *Tenm2*. In the Lean line genes *Csmd3*, *Erbb4*, and *Inpp4b* had the most SNPs. Meanwhile, in the Fat line, these were *Skint5*, *Exoc4*, and *Galnt2l*. Other genes in the Fat line include *Adcy2*, *Cpq*, *Ctnna3*, *Dcc*, *Fgf14*, *Gm37013*, *Hdac9*, *Lhfpl3*, *Mast4*, *Mctp1*, *Ndst4*, *Skint6*, *Slc14a2*, *Slc9a9*, *Smyd3*, *Sntg1*, *Sox5*, and *Trpm3*, and in the Lean line *5730522E02Rik*, *Atrnl1*, *Cdkal1*, *Diaph3*, *Edil3*, *Fstl5*, *Gabrb3*, *Gmds*, *Hcn1*, *Hs3st4*, *Immp2l*, *Oca2*, *Pak5*, *Pcsk5*, *Prkn*, *Prr16*, *Slc7a11*, *Tenm4*, and *Zfp536* (Supplementary Table S3).

Considering the relative number of SNPs per gene (SNPs/bp), 30 genes (Lean: 12, Fat: 18) had at least four SNPs per 100 bp. Compared to the genes with the highest absolute number of SNPs, the SNP density was highest in pseudogenes and various types of regulatory RNAs. In more detail: gene segment (*Ighd2-6*), miRNAs (*Gm23063*, *Mir3086*, *Mir7237*), miscellaneous RNA (*Gm25403*), pseudogenes (*AC152418.1*, *Ear-ps10*, *Gm15115*, *Gm34658*, *Gm37489*, *Gm43943*, *Gm47555*, *Gm4873*, *Gm49055*, *Gm9002*, *Olfr1139-ps1*, *S100a11-ps*), pseudogenic gene segments (*Gm43220*, *Igkv1-136*), RNase P RNA gene (*Rprl1*), rRNAs (*Gm23668*, n-R5s3), snoRNA gene (*Gm24127*), and snRNA genes (*Gm27385*, *Gm26449*, *Gm25785*, *Gm24725*, *Gm24582*, *Gm23511*, *Gm22828*) (Supplementary Table S4).

For two miRNAs, *Mir7237* and *Mir3086*, we explored whether SNPs fall into their seed region, altering their target sequence recognition. Whereas no SNP was within the for *Mir7237* seed region, the SNP rs248726381 (T/C) in the Lean line fells into the seed region of *mmu-miR-3086-3p* (CCAAUGA◊ CCAAUG**G**) (Fig. S6a). Several biological processes in the Lean line could be affected due to polymorphism in the seed region of this miRNA caused by rs248726381, including histone and protein acetylation (Fig. S6b). Further enrichment analysis of *mmu-miR-3086-3p* target genes revealed that, in addition to amino acid metabolism and the nervous system, the target genes also participate in fatty acid biosynthesis (Supplementary Fig. S6c).

### GERP scores of line-specific SNPs

GERP score of SNPs was then retrieved to reveal potentially functional SNPs. Most SNPs have GERP score between − 1 and 1, however, 276,397 SNPs have GERP score above 2, 1,369 SNPs above 4, and 125 SNPs above 6 (Supplementary Fig. 7). The latter group include 13 SNPs in regulatory elements within or close to 14 genes, and 6 SNPs with predicted moderate or high impact on 6 genes.

### Variant biotype of line-specific SNPs

**Fig. 3:**
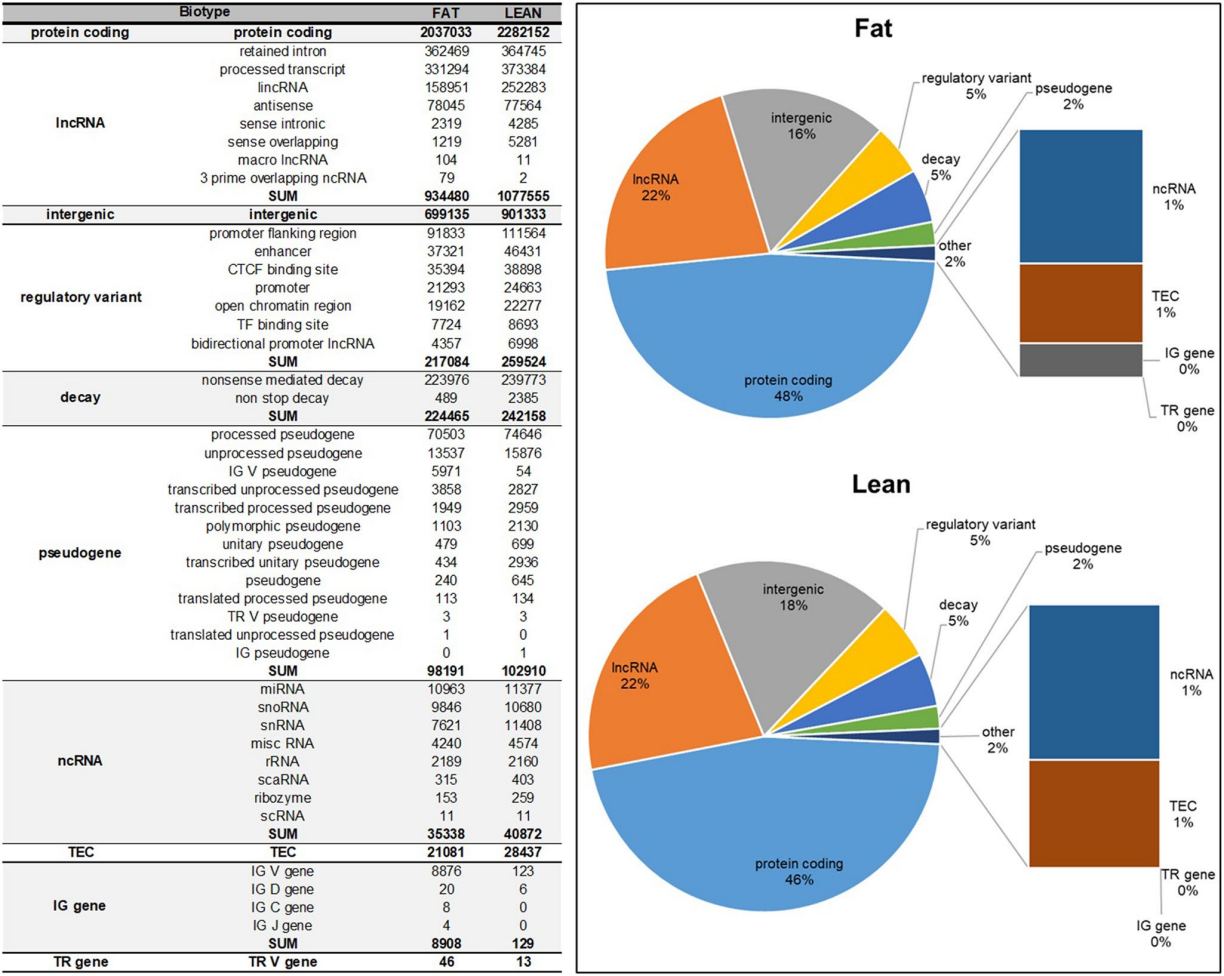
Biotypes and share of line-specific variants identified in the Lean and Fat mouse selection lines

The most represented line-specific variant biotype was protein coding, followed by lncRNA and intergenic variants. Interestingly, the largest relative difference in the number of SNPs between the lines was IG gene biotype (especially IG V gene) with 8908 SNPs identified in the Fat line and only 129 in the Lean line (Fig. 3).

### Variant consequences of line-specific SNPs

In both lines, most of the SNPs were intronic variants, followed by intergenic, non-coding transcript, downstream, and upstream variants (Supplementary Fig. S8). In total, 887,371 (Lean) and 778,626 (Fat) line-specific SNPs were located within 13,120 and 13,094 genes, representing 45% and 47% of all the SNPs, respectively.

In the Lean line, synonymous and missense variants accounted for 0.32% and 0.15%, respectively, while in the Fat line they accounted for 0.36% and 0.19% of all SNPs. Among all the missense variants, predicted deleterious variants represented 17.4% and 17.7% in the Lean and Fat lines, respectively. In the Lean line, they were mainly located on chromosomes 2, 7, and 9, while in the Fat line they were found on chromosomes 7, 4, and 6 (Supplementary Fig. S9).

We next investigated if predicted DMVs were located within genes related to obesity (abnormal adipose tissue amount - IMPC). In both mice, line-specific DMVs within several obesity-related genes were identified (Fat: 11, Lean: 12). In addition, 24 genes had shared DMVs (Supplementary Table S5). Among the DMVs, nine SNPs within nine genes were newly identified: 12_13433013_T/A in *Nbas*, 6_128327592_C/T in *Tulp3*, and 7_77124609_C/T in *Agbl1* of the Fat line, 15_48791841_G/C in *Csmd3*, 18_59409565_G/T in *Chsy3*, and 6_95117339_C/T in *Kbtbd8* of the Lean line, and 11_87874953_A/G in *Epx*, 16_35824901_G/A in *Hspbap1*, and 8_84872251_G/A in *Syce2* of both lines. Other obesity genes in the Fat line include *2210408I21Rik*, *Cep250*, *Fam81b*, *Il6st*, *Mamld1*, *Nbas*, *Pth1r*, *Sema4d*, and *Slco1b2*. Meanwhile, in the Lean line these are *Alg8*, *Alpk2*, *Aspm*, *D430041D05Rik*, *D630045J12Rik*, *Dock9*, *Gpr15*, *Phldb1*, and *Zfp462*. Worth mentioning is *Csmd3*, a gene with the highest number of SNPs in the Lean line (shown in Supplementary Table S3).

Enrichment analysis revealed that in both lines the genes with line-specific DMVs are involved in the following pathways: graft-versus-host disease, type I diabetes mellitus, and allograft rejection. Other pathways in the Fat line included antigen processing and presentation, serotonergic synapse, viral myocarditis, and asthma. In contrast, genes with Lean line-specific DMVs were involved in cell adhesion molecules, autoimmune thyroid disease, and inflammatory bowel disease. Interestingly, in both lines, the genes mostly related to pathways potentially involved in food perception attained the lowest *p*-value; olfactory transduction in the Lean line and taste transduction in the Fat line (Supplementary Fig. S10).

For the taste and olfactory transduction pathways, we then explored whether the genes with DMVs map to previously identified obesity/leanness QTLs. While *Plcb2*, a gene involved in the taste transduction, locate within the *Fob1* QTL, 26 out of 75 genes of olfactory transduction are within *Fob1* QTL: *Or4b1d*, *Or5d47*, *Or5aq7*, *Or9g4b*, *Or5m12*, *Or5m13b*, *Or8k1*, *Or8k20*, *Or8k24*, *Or8k28*, *Or8k32*, *Or8h9*, *Or8h10*, *Or5j1*, *Or5aq6*, *Or5w10*, *Or10ag59*, *Or8w1*, *Or5w1b*, *Or5w13*, *Or5w15*, *Or5w17*, *Or4a2*, *Or4a69, Or4a74*, and *Or4f57*.

We than analyzed the density of line-specific missense variants in protein-coding transcripts to identify proteins exhibiting significant differences between the lines. We found 38 transcripts (Fat line: 28, Lean lin: 10) that had an average density of missense variants at intervals of fewer than 25 amino acids. These transcripts correspond to 32 genes (Fat line: 21, Lean line: 10, and 1 common to both). Notably, the genes associated with the highest density of missense variants in the Fat line include *Cx3cl1*, *Nlrp1b*, *Tas2r136*, *Cd22*, and *Or5p58*. Meanwhile, in the Lean line these are *Hbb-bh2*, *Kcnmb2*, *Ang5*, *Or5w17*, and *Cbr1b*, as detailed in Supplementary Table S6. Interestingly, *Hamp2* appears in the top five for both lines, albeit represented by different transcripts (ENSMUST00000205641 in the Fat line and ENSMUST00000109753 in the Lean line). Additionally, the genes *Skint5* and *Macrod2* were identified as having a notably high number of variants, as shown in Supplementary Table S3.

Eighteen of these genes are involved in 35 different KEGG pathways (Supplementary Table S7). However, three genes are involved in the majority of these pathways: *Cx3cl1* (immune and inflammatory reactions) and *H2-Aa* and *H2-Ab1* (various disease, immune system, and type I diabetes mellitus). Worth mentioning are eight genes potentially involved in food perception, among which *Or5w17* and *Or8k28* are within FOB QTL *Fob1*. Another gene in FOB QTL is *Tmsb15b1*. In addition, *Kcnmb2* and *Nlrp1b* are involved in insulin secretion and NOD-like receptor signalling pathway, respectively.

### Annotation of candidate genes

Genes with regulatory variants and variants with moderate or high impact with the GERP score above 6 were further analysed and their annotation related to obesity retrieved from various sources. In total, 14 genes have 13 regulatory SNPs, and 6 genes have 6 SNPs with predicted moderate or high impact. These 20 genes include three lncRNAs (*4930441H08Rik*, *4930595O18Rik*, *Gm36633*), one polymorphic pseudogene (*Or56b2j*), 15 protein coding genes (*Aff3*, *Angpt1*, *Atpsckmt*, *Cpped1*, *Erc2*, *Gfra1*, *Fam237b*, *Mast4*, *Pced1a*, *Prr5l*, *Serpine2*, *Tecrl*, *Tmem132d*, *Trim24*, *Zfp536*), and one pseudogene (*Gm17131*). Two genes are within FOB QTL (*Pced1a* and *Prr5l*). *Gfra1*, *Or56b2j*, *Serpine2*, and *Tecrl* were the only genes previously annotated with obesity-related traits. In addition to the above-mentioned *Gfra1*, *Prr5l*, and *Serpine2*, differential expression was also measured for *Angpt1*, *Tmem132d*, and *Trim24*.

**Table 1:** Prioritization of candidate genes. Genes carrying SNPs with GERP score above 6 located in regulatory regions or having a predicted moderate or high impact on protein function

Gene symbol	Gene name	Gene biotype	Regulatory SNP	Moderate or high impact SNPs	FOB QTL	IMPC^2^	Gene ontology^3^	Mammalian phenotype^4^	Human disease^5^	Expression^6^	High number of SNPs^7^	Validated by MGI^8^
*4930441H08Rik*	RIKEN cDNA 4930441H08 gene	lncRNA gene	*L:1 PFR, F:0*	*-*								✓
*4930595O18Rik*	RIKEN cDNA 4930595O18 gene	lncRNA gene	*L:1 PFR, F:0*	*-*								
*Aff3*	AF4/FMR2 family, member 3	protein coding gene	*-*	*L:1, F:0*								✓
*Angpt1*	angiopoietin 1	protein coding gene	*L:1 TFBS, F:0*	*-*						✓		
*Atpsckmt*	ATP synthase C subunit lysine N-methyltransferase	protein coding gene	*L:0, F:1 TFBS*	*-*								
*Cpped1*	calcineurin-like phosphoesterase domain containing 1	protein coding gene	*L:1 PFR, F:0*	*-*								
*Erc2*	ELKS/RAB6-interacting/CAST family member 2	protein coding gene	*L:0, F:1 E*	*-*								
*Gfra1*	glial cell line derived neurotrophic factor family receptor alpha 1	protein coding gene	*L:1 E, F:0*	*-*				✓		✓		
*Gm17131*	predicted gene 17,131	pseudogene	*L:0, F:1 PFR and CTCFBS*	*-*								
*Gm36633*	predicted gene, 36,633	lncRNA gene	*L:1 PFR, F:0*	*-*								✓
*Fam237b*	family with sequence similarity 237, member B	protein coding gene	*-*	*L:1, F:0*								✓
*Mast4*	microtubule associated serine/threonine kinase family member 4	protein coding gene	*L:1 PFR, F:1 PFR*	*-*							✓	
*Or56b2j*	olfactory receptor family 56 subfamily B member 2 J	polymorphic pseudogene	*-*	*L:0, F:1*			✓					
*Pced1a*	PC-esterase domain containing 1 A	protein coding gene	*-*	*L:0, F:1*	✓							✓
*Prr5l*	proline rich 5 like	protein coding gene	*L:1 PFR and OCR, F:0*	*-*	✓					✓		
*Serpine2*	serine (or cysteine) peptidase inhibitor, clade E, member 2	protein coding gene	*L:1 P, F:0*	*-*			✓			✓		
*Tecrl*	trans-2,3-enoyl-CoA reductase-like	protein coding gene	*-*	*L:0, F:1*			✓					✓
*Tmem132d*	transmembrane protein 132D	protein coding gene	*L:0, F:1 PFR and CTCFBS*	*-*						✓		
*Trim24*	tripartite motif-containing 24	protein coding gene	*-*	*L:1, F:0*						✓		
*Zfp536*	zinc finger protein 536	protein coding gene	*L:1 PFR, F:0*	*-*							✓	✓

1 - E: enhancer, CTCFBS: CTCF binding site, P: promoter, PFR: promoter flanking region, TFBS: transcription factor binding site

2 - search term: abnormal adipose tissue amount

3 - search keywords: adipo, cholesterol, digestion, eating, energy, fat, feeding, glucose, glycogen, insulin, lean, leptin, lipid, lipoprotein, obese, olfactory, phagy, smell, storage, taste, thermogenesis

4 - search keywords: adipose, cholesterol, digestion, eating, energy, fat, fatigue, feeding, glucose, glycogen, insulin, lean, leptin, lipid, lipoprotein, obese, olfactory, phagy, smell, storage, taste, thermogenesis

5 - search keywords: cardio, diabetes, dystrophy, eating, feeding, glycemia, glycogen, heart, hypertension, insulin, lean, leptin, lipidemia, lipodystrophy, liver, muscular, obesity, olfactory, osteoarthritis, phagy, smell, taste, thermogenesis

6 - Differential gene expression (Microarray or RNA-seq)

7 - Supplementary Table S3

8 - SNP is present in Fat line and obese mouse NZO/HlLtJ or present in the Lean line and obesity resistant A/J mouse line

**Fig. 4:**
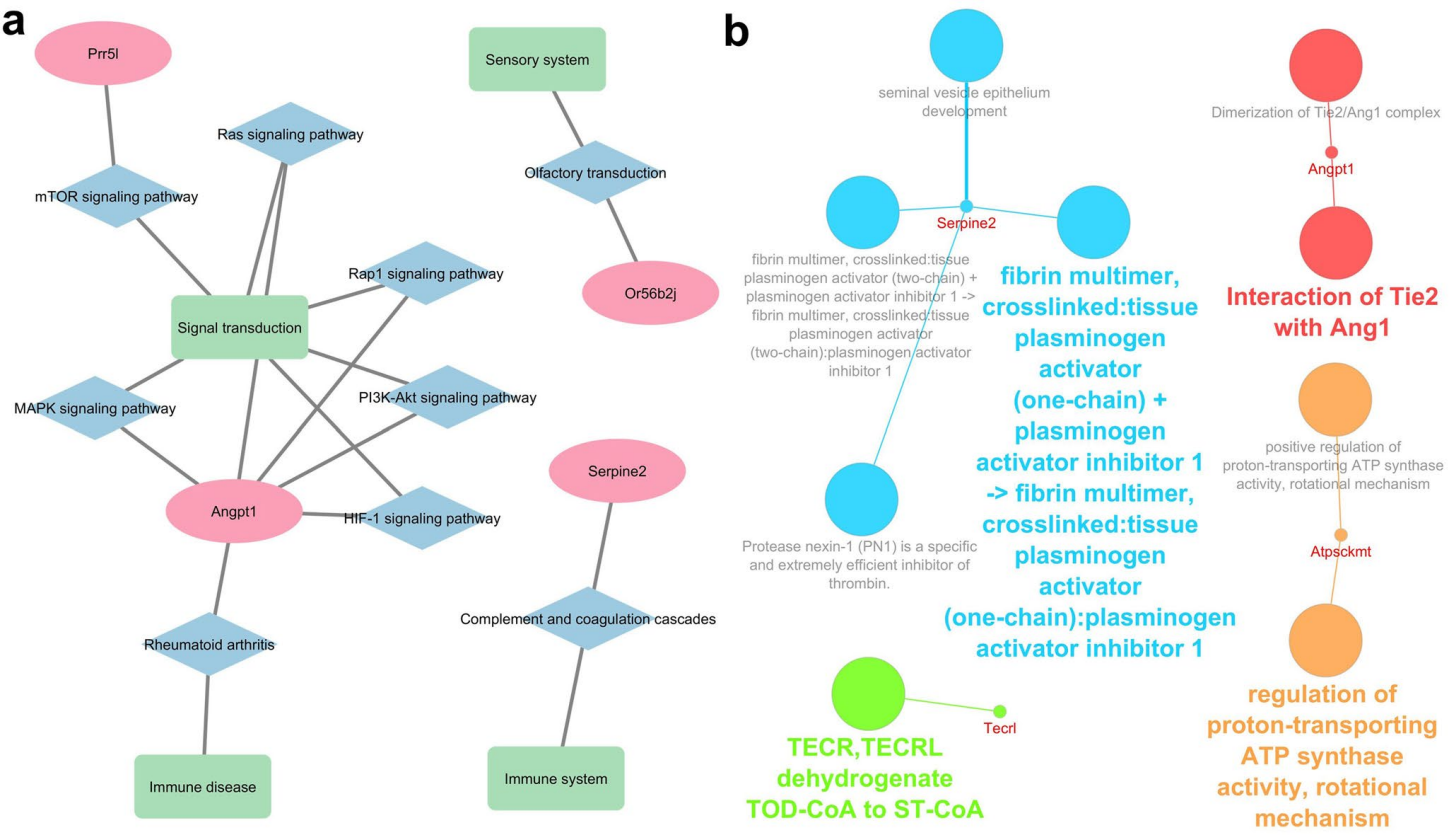
Biological function of the genes carrying SNPs with GERP score above 6 located in regulatory regions or having predicted moderate or high impact on protein function identified in the Fat and Lean mouse selection lines. **a**) KEGG ontology terms, **b**) biological function of identified genes

Out of 20 genes only four are annotated in the KEGG database: *Angpt1* being involved in various signalling pathways, *Prr5l* in mTOR signalling pathway, *Serpine2* in immune system, and *Or56b2j* in sensory (olfactory) system (Fig. 4a). The signalling function of ANGPT1 protein might be due to the interaction with TIE2 (TEK receptor tyrosine kinase). ATPSCKMT is a positive regulator v ATP synthase activity, and TECRL catalyses trans-octadec-2-enoyl-CoA to stearyl-CoA (Fig. 4b).

Finally, the potential involvement of 19 identified SNPs within 20 genes in obesity was validated by comparing alleles between our mice lines and obesity prone NZO/HlLtJ and obesity resistant A/J strain. The analysis revealed four missense SNPs and 2 regulatory variants within 7 genes (*4930441H08Rik*, *Aff3*, *Fam237b*, *Gm36633*, *Pced1a*, *Tecrl*, *Zfp536*) (Table 2).

**Table 2:** Allele comparison between fat lines (our Fat line and NZO/HlLtJ) and lean lines (our Lean line and A/J)

Gene symbol	SNP	PHENOTYPE				Consequence
		FAT		LEAN		
		Fat (FLI) line	NZO/HILtJ	Lean (FHI) line	A/J	
*Pced1a*	rs33645896	**A**	**A**	G	G	missense variant
*Tecrl*	rs31632835	**C**	**C**	A	A	missense variant
*Aff3*	rs30511899	T	T	**G**	**G**	missense variant
*Fam237b*	rs31007064	T	T	**C**	**C**	missense variant
*Zfp536*	rs46750862	G	G	**A**	**A**	promoter flanking region
*4930441H08Rik*, *Gm36633*	rs31031645	A	A	**G**	**G**	promoter flanking region

## Discussion

Obesity has become one of the serious health challenges worldwide. Since 1975, the number of obese individuals has tripled (Vaamonde and Álvarez-Món 2020). With advances in DNA/RNA sequencing technologies, several genes and genetic variants responsible for monogenic and polygenic forms of obesity have been identified, as well as strategies for obesity treatment (Hinney et al. 2010; Ginete et al. 2021; Loos and Yeo 2022). In this study, we delve into this intricate genetic landscape using WGS to explore SNPs in two distinct unique mouse models, representing the polygenic nature of obesity and its counterpart, leanness. The study not only expands our understanding of the genetic basis of obesity and proposes several candidate genes but also paves the way for developing novel or more effective treatments and interventions.

The WGS analysis identified 6,309,795 SNPs (Lean: 4,651,068, Fat: 4,320,310, Both: 2,661,583), of which 1,303,138 were newly identified, primarily involved in localization, response to stimulus, and signalling. The highest number of SNPs was identified within protein-coding genes, two of which had more than 3000 line-specific SNPs in both lines, *Macrod2* (mono-ADP ribosylhydrolase 2) and *Tenm2* (teneurin transmembrane protein 2). In the Lean line, *Csmd3* (CUB and Sushi multiple domains 3), *Erbb4* (erb-b2 receptor tyrosine kinase 4), and *Inpp4b* (inositol polyphosphate-4-phosphatase, type II) genes had the most SNPs, while in the Fat line, these were *Skint5* (selection and upkeep of intraepithelial T cells 5), *Exoc4* (exocyst complex component 4), and *Galnt2l* (polypeptide N-acetylgalactosaminyltransferase 2-like). Both *Macrod2* and *Tenm2* have been implicated in adipogenesis (Tews et al. 2017; Chang et al. 2018). Significant associations were observed between morbid obese Han Chinese and *CSMD3* (obesity related, IMPC) and *ERBB4* (Chiang et al. 2019). *INPP4B* protects against metabolic syndrome and associated disorders (Zhang et al. 2021) and *EXOC4* is involved in insulin-stimulated glucose transport and is associated with type II diabetes and fasting glucose (Laramie et al. 2008). There is no scientific literature available for the mouse gene *Galnt2l* and no ortholog has been detected so far in human. However, according to the Ensembl database *Galnt2l* shares 98.69% similarity with *Galnt2*. Loss of function of *GALNT2* lowers high-density lipoproteins in different species (Khetarpal et al. 2016) and is associated with metabolic diseases, including obesity (Antonucci et al. 2022). Worth mentioning are also two genes that are located within the *Fob2* QTL on chromosome 12; *Hdac9* (histone deacetylase 9) and *Immp2l* (IMP2 inner mitochondrial membrane peptidase-like (*S. cerevisiae*). Global deletion of *Hdac9* protects against high-fat diet (HFD) induced obesity and metabolic disease in mice (Chatterjee et al. 2014), and *Immp2l* is associated with food intake (Han et al. 2013). Another potential candidate gene is *Pcsk5* (proprotein convertase subtilisin/kexin type 5). In total, 4587 SNPs were identified in the gene (Lean: 3497, Fat: 617, Both: 473). More importantly, 26 SNPs in the Fat line are shared with other obese mice strains (AKR/J, NZO/HILtJ) but not with the lean lines (A/J, BALB/cJ, FVB/NJ, CAST/EiJ). *PCSK5* locus was associated with serum low- and high-density lipoprotein (LDL and HDL) and cholesterol levels (Iatan et al. 2009; De Vries et al. 2019). Moreover, PCSK5 might be a potential target to lower LDL levels (De Vries et al. 2019) and valuable for the ANGPTL4 (angiopoietin like 4)-mediated target therapy of diabetes and metabolic syndrome (Li and Teng 2014). Therefore, the *Pcsk5* might be a potential obesity candidate gene to be explored in the future.

Considering the relative number of SNPs per gene (SNPs/bp), the SNP density was the highest in pseudogenes and various types of regulatory RNAs, which is consistent with previous findings (Balasubramanian et al. 2002; Yang et al. 2017b). This could be because pseudogenes and regulatory RNAs are subjected to less selection pressure at the sequence level compared to the protein-coding genes that are evolutionarily more highly conserved (Balasubramanian et al. 2002; Yang et al. 2017b). Although having been labelled as “junk” DNA, the potential functions of pseudogenes in the expression regulation of protein-coding genes have been described, mainly acting as small interfering RNAs or miRNA scavengers (Pink et al. 2011). Among the genes with at least four line-specific SNPs per 100 bp identified in the Lean and Fat lines, three genes were located in the *Fob2* QTL interval: snRNA *Gm26449*, and pseudogenes *S100a11-ps* (S100 calcium binding protein A11, pseudogene) and *Gm47555*, and one in the *Fob1* QTL interval: pseudogene *Olfr1139-ps1* (olfactory receptor 1139, pseudogene 1). Based on the Ensembl database, *Gm26449* is part of the spliceosomal small nuclear ribonucleoprotein complexes, and an orthologue of U6, suggesting differential pre-mRNA processing between the two lines. The function of the other three genes is currently unknown.

The SNPs in miRNAs may affect the miRNA functions by altering miRNA maturation, structure, expression, and target binding and, consequently, contribute to phenotype and disease susceptibility (Titov and Vorozheykin 2018). In the present study, the SNP rs248726381 identified in the Lean line locates within the seed region of *mmu-miR-3086-3p*, potentially affecting histone and protein acetylation modifications. Both, histone and protein acetylation regulate obesity (Xu et al. 2019). The genes targeted by *mmu-miR-3086-3p* are also involved in fatty acid biosynthesis, making *mmu-miR-3086-3p* and its polymorphism of interest for the future research. In our previous study (Kunej et al. 2010), a SNP within the seed region of obesity-related *mmu-miR-717* was identified in the lean mouse strain 129/Sv. The presence of SNP (rs30372501) within the seed region of this miRNA was also identified in the present study, however, it is present in both lines. Future work might focus on the potential polymorphisms within the binding sites on its target genes.

In addition to the above, identified SNPs and indels in the introns of the pseudogene *Mid1-ps1* (midline 1, pseudogene 1) and the long non-coding RNA *Gm47283* are also worth mentioning. For the *Mid1-ps1*, one SNP was identified in the Lean line and four were shared by both. For the *Gm47283*, four SNPs and several indels were identified in both lines, however, nine were specific to the Lean line. Interestingly, all variants in this gene were located within the same intron and overlap various regulatory elements (Supplementary Fig. S11). Introns play an important role in gene regulation and expression, and therefore SNPs in introns can potentially affect the phenotype and cause disease (Salih et al. 2021). The two genes, *Mid1-ps1* and *Gm47283* (also known as *Erdr1* (erythroid differentiation regulator 1) (Merkenschlager et al. 2019), are within the pseudoautosomal region (PAR) of the sex chromosome Y (Yamazaki et al. 2021). Although PAR genes should behave like autosomal genes, the expression of both genes was unexpectedly higher in the XY oocytes than in XX and XO oocytes (Yamazaki et al. 2021). Importantly, the Fat and Lean lines average less than four pups per litter with other reproductive parameters (i.e. time from mate to litter) also being at the lower end compared to other standard inbred strains. Our preliminary analysis (Pirman et al. 2022) suggests that a large negative effect on reproduction may come from the males. Since *Erdr1* knockout has been shown to result in the embryonic lethality in mice (Zuo et al. 2017), and *Mid1* regulates mTOR (mammalian target of rapamycin) signalling (Liu et al. 2011), which is required for embryonic development (Gangloff et al. 2004), the identified genetic variants on chromosome Y in genes *Gm47283* and *Mid1-ps1* may be functionally important for the reproductive performance of the two lines and are worth exploring in further functional analyses.

In total, 2826 predicted DMVs have been identified (Lean: 843, Fat: 991, Both: 992), which are also present in several obesity-related genes (IMPC). Among these are nine novel DMVs within nine genes: *Agbl1* (ATP/GTP binding protein-like 1), *Chsy3* (chondroitin sulfate synthase 3), *Csmd3* (the aforementioned gene with the most line-specific SNPs), *Nbas* (neuroblastoma amplified sequence), *Epx* (eosinophil peroxidase), *Hspbap1* (Hspb associated protein 1), *Kbtbd8* (kelch repeat and BTB (POZ) domain containing 8), *Syce2* (synaptonemal complex central element protein 2), and *Tulp3* (tubby-like protein 3). Moreover, DMVs were also found within the “obesity” genes located in FOB QTLs: *D430041D05Rik* and *Frmd5* (FERM domain containing 5) in *Fob1*, the aforementioned *Nbas* in *Fob2*, and *Mamld1* (mastermind-like domain containing 1) in *Fob4*. Human AGBL1 was associated with levels of circulating adiponectin, a hormone released from adipose tissue that affects insulin sensitivity and inflammatory patterns, and linked to body mass index, visceral fat, and risk of type 2 diabetes mellitus (Hasegawa et al. 2022). EPX has both anti-inflammatory and pro-inflammatory effects and its expression increases in obesity (Yi et al. 2021). A histone demethylase HSPBAP1 may be involved in regulating stress responses in cells by inhibiting HSP27 (Cloos et al. 2008), which has been suggested to prevent obesity-induced insulin resistance (McCarty 2006). KBTBD8, a gene involved in cell mitosis, has been associated with growth, particularly idiopathic short stature in a Korean population (Kim et al. 2010). Importantly, the novel DMV 6_95117339_C/T in the Lean line is also present in other lean mice strains (A/J, BALB/cJ, FVB/NJ, CAST/EiJ) but not in the obese lines (AKR/J, NZO/HILtJ), suggesting this DMV to be functionally important and may contribute to the lean phenotype. Recently, TULP3 has been demonstrated to be required for the transport of G protein-coupled receptors to primary cilia, and its knockdown impaired ciliary FFAR4 (free fatty acid receptor 4) and PTGER4 (prostaglandin e receptor 4) localization and regulated glucagon or insulin secretion (Wu et al. 2021). As for the genes in FOB QTLs, variant rs524908 in human *FRMD5* was linked to the serum lipid profile, such as low-density lipoprotein cholesterol and apolipoprotein B levels (Guo et al. 2017). Considering the previous paragraph, it is important to note that *Mamld1* mediates testosterone production for male sex development (Nakamura et al. 2011), and pathogenic variants within *MAMLD1* are associated with 46,XY differences/disorders of sex development, with genital abnormalities at birth and possibly associated with age-dependent deterioration of testicular function (Miyado et al. 2022). In addition, *MAMLD1* is one of the candidate genes for early-onset obesity (Pettersson et al. 2017), linking the genetic basis of obesity with reduced reproductive performance.

KEGG pathway enrichment analysis revealed that genes carrying DMVs are enriched in various pathways. While the pathways graft-versus-host disease, type I diabetes mellitus, and allograft rejection were common to both lines, other, line-specific pathways were also identified, such as taste transduction, antigen processing and presentation, and serotonergic synapse for the Fat line, and olfactory transduction, cell adhesion molecules, autoimmune thyroid disease, and inflammatory bowel disease for the Lean line. These results suggest that the two lines have different taste, olfaction, and immune signalling. For example, graft-versus-host disease and allograft rejection pathways are enhanced by obesity (Molinero et al. 2016; Khuat et al. 2020). Given the available literature (Lei et al. 2016; Zuo and Ng 2018; Khuat et al. 2020), the two pathways and the inflammatory bowel disease pathway could indicate that the Fat and Lean lines have distinct gut microbiomes and environments that may affect nutrient metabolism and energy expenditure (Aoun et al. 2020). Moreover, the enrichment of genes carrying DMVs in the antigen processing and presentation pathway of the Fat line is consistent with the notion that the Fat line has more SNPs in genes encoding different immunoglobulin chains compared to the Lean line. According to Winer et al. (2014) and Majdoubi et al. (2016) (Winer et al. 2014; Majdoubi et al. 2016) these findings may indicate alterations in B cell function, inflammation, and insulin resistance in the Fat line. Interestingly, the pathways involved in food perception were the most enriched in both lines; taste transduction in the Fat line, and olfactory transduction in the Lean line. Recent studies have shown that food perception alone can induce metabolic changes (Brandt et al. 2018; Kaplan et al. 2018). Hypothalamus is at the crossroad of olfactory and gustatory sensing and regulation of food intake and energy homeostasis (Chao et al. 2016; Faour et al. 2022). In our preliminary study on the hypothalamic transcriptome difference of the two mouse lines fed by a normal chow 148 differentially expressed genes (adjusted *p* < 0.05, fold change ≥ 1.5) were identified, including four involved in olfactory perception: *Or10h1b*, *Or5v1b*, *Or52n4*, and *Or5m3b* (unpublished data). In this study, 26 out of 75 genes of olfactory transduction carrying DMVs in the Lean line are within *Fob1* QTL (Horvat et al. 2000). Moreover, interestingly, the Fat line carries DMVs in 12 receptors for bitter taste and downstream signalling component for bitter/sweet/umami taste, *Plcb2* (phospholipase C, beta 2) (Behrens and Meyerhof 2006). Furthermore, a novel DMV 17_37050960_C/T in the Fat line is also within *Gabbr1* (gamma-aminobutyric acid (GABA) B receptor, 1), a gene associated with conditioned taste aversion (Jacobson et al. 2006). In addition, our whole transcript termini site sequencing (Mikec et al. 2023) revealed differential usage of polyadenylation sites in the 3’UTR of this gene between the two lines. In short, these results are suggesting that food perception might contribute to the obese/lean phenotype of our mouse models and require further functional examinations to identify the candidate genes.

Thirty-eight transcripts (Fat line: 28, Lean line: 10) have an average density of missense variants at intervals of fewer than 25 amino acids. Among the genes coding for these proteins are also *Or5w17*, *Or8k28*, and *Tmsb15b1* located within FOB QTLs. The olfactory receptors have already been discussed above. Meanwhile, thymosin beta 15 (*Tmsb15b1*) plays an important role in maintaining the dynamic balance of actin, angiogenesis, axonal formation, and wound healing. Recent evidence suggests it to participate in the differentiation and function of thymic epithelial cells, which play an important role in in the thymus response to external stimuli and production of mature T cells (Xu et al. 2022). According to Park and Shastri (2022), the potential imbalance of thymocyte differentiation in the Fat line may contribute to its obese phenotype. Another gene also involved in angiogenesis is *Ang5* (angiogenin, ribonuclease A family, member 5). Angiogenic factors, including angiogenin, were associated with adipose tissue remodelling and development of obesity (Kurki et al. 2012).

Several other genes with high density of missense variants are involved in inflammation and immune response, such as *Cx3cl1*, *H2-Aa*, *H2-Ab1*, *Fkbpl*, *Mok*, *Cd22*, *Nlrp1b* and members of selection and upkeep of intraepithelial T cells (*Skint1*, *Skint5*, *Skint8*). For example, microglial CX3CR1 (C-X3-C motif chemokine ligand 1) signalling determine obesity susceptibility in mice (Dorfman et al. 2017) and its deficiency induces inflammation and insulin resistance in adipose tissue (Nagashimada et al. 2021). In addition, this signalling affects hypothalamic POMC neuronal excitability and melanocortin system activity (Banerjee et al. 2022). These results make *Cx3cr* and its polymorphisms in the Fat line strong obesity candidates. Meanwhile, CD22 (CD22 antigen) was suggested as potential therapeutic target in hypertensive overweight and obese adults (Liao et al. 2022).

WGS revealed high density of missense variants in different transcripts of *Hamp2* (hepcidin antimicrobial peptide 2) between the two lines. Hepcidin (a master regulator of iron metabolism) induction by human symbiont *Bacteroides thetaiotaomicron* was demonstrated to increase fat deposition by supressing the aforementioned lipoprotein lipase inhibitor ANGPTL4 in the small intestine (Cho et al. 2022). Hepcidin also affects iron acquisition for hemoglobin synthesis (González-Domínguez et al. 2020). Interestingly, in the Lean line, the highest density of missense variants was observed in *Hbb-bh2* (hemoglobin beta, bh2), suggesting distinct iron metabolism between the two lines may contribute to their divergent phenotypes. In the Fat line, two transcripts of *Nlrp1b* (NLR family, pyrin domain containing 1B) were among those with the highest density of missense variants. Mice deactivated for NLRP1 inflammasome were shown to spontaneously develop obesity due to the decreased IL-18 production and lipolysis (Murphy et al. 2016). A metabolic enzyme CBR1B (carbonyl reductase 1B) amplifies glucocorticoid action in adipose tissue and impairs glucose tolerance in mice (Bell et al. 2021). Considering missense variants within *Cbr1b* were identified in the Lean line, *Cbr1b* deficiency may contribute to the lean phenotype.

The GERP score is defined as the reduction in the number of substitutions in a multi-species sequence alignment compared to what’s expected under neutral evolution. High GERP scores suggest that mutations in these conserved areas are likely harmful, impacting the species’ genetic health, and GERP scores between 4 and 6 were defined to have “large” deleterious effects (Huber et al. 2020). We annotated the genes with regulatory variants and variants with moderate or high impact with the GERP score above six (Table 1). In total, 14 genes have 13 regulatory SNPs, and 6 genes have 6 SNPs with predicted moderate or high impact. These 20 genes are *4930441H08Rik*, *4930595O18Rik*, *Aff3*, *Angpt1*, *Atpsckmt*, *Cpped1*, *Erc2*, *Gfra1*, *Gm17131*, *Gm36633*, *Fam237b*, *Mast4*, *Or56b2j*, *Pced1a*, *Prr5l*, *Serpine2*, *Tecrl*, *Tmem132d*, *Trim24*, and *Zfp536*. Of these, only four have been annotated with obesity-related ontologies: *Gfra1*, *Or56b2j*, *Serpine2*, and *Tecrl*. The remaining 16 might represent novel obesity candidates, especially *4930441H08Rik*, *Aff3*, *Fam237b*, *Gm36633*, *Pced1a*, and *Zfp536* with alleles differing between fat (our Fat line and NZO/HlLtJ) and lean lines (our Lean line and A/J).

GFRA1 receptor (glial cell line derived neurotrophic factor family receptor alpha 1) in human was identified as the mediator for the anorectic effects of the protein GDF15 (growth differentiation factor 15), which has shown potential in decreasing food intake and body weight in obese animal models (Yang et al. 2017a), GDF15–GFRAL system acting independently of the GLP1 (glucagon-like peptide-1) and leptin pathways (Cimino et al. 2017). No relevant scientific data are available for *Or56b2j* (olfactory receptor family 56 subfamily B member 2 J), however, a study by Choquette et al. (2012) suggests that variations in human olfactory receptor genes can influence eating behaviours and adiposity. In addition, deletion of olfactory receptor-rich region 11q11 was identified as a risk factor for obesity (Diels et al. 2020). SERPINE2 (serine (or cysteine) peptidase inhibitor, clade E, member 2) pathway is evolutionary conserved and involved in tumorigenesis (Shen et al. 2017). Moreover, *SERPINE2* was one of the genes most differentially methylated and expressed between non-obese and obese individuals in human adipose tissue (Keller et al. 2017) and recognized as a potential biomarker of obesity-induced alteration in placenta development (Altmäe et al. 2017). TECRL (trans-2,3-enoyl-CoA reductase-like) deficiency is associated with aberrant mitochondrial function in cardiomyocytes (Hou et al. 2022), due to the alterations in fatty acid metabolism and consequent electrophysiological anomalies (Gelinas et al. 2017). *TECRL* was identified as a new life-threatening inherited arrhythmia gene associated with overlapping clinical features of both long QT syndrome and catecholaminergic polymorphic ventricular tachycardia (Devalla et al. 2016). A missense variant rs31632835 in the Fat line (as well in NZO/HILtJ) may therefore account for its lower physical activity compared to the Lean line observed by Simončič et al. (2008).

In addition to *Tecrl*, MGI validation uncovered other genes: *4930441H08Rik*, *Aff3*, *Fam237b*, *Gm36633*, *Pced1a*, and *Zfp536*. No scientific literature is available for *4930441H08Rik*. However, among the top ten predicted targets of *4930441H08Rik* by LncRRIsearch tool include genes potentially involved in obesity: *Hal* (histidine ammonia lyase) (Böhm et al. 2014; Ren et al. 2016), *H19* (H19, imprinted maternally expressed transcript) (Schmidt et al. 2018; Wang et al. 2021a), *Igf2* (insulin-like growth factor 2) (Queiroz et al. 2015; Ács et al. 2017), and lncRNA *Nova2os* (NOVA alternative splicing regulator 2, opposite strand sequence) (Mikec et al. 2022). *AFF3* (AF4/FMR2 family, member 3) was recognized as a master regulator of metabolic inflexibility in type 2 diabetes (Son et al. 2019) and was associated with serum lipid alterations and blood triglyceride levels (Li et al. 2015). Perhaps very interesting is *Fam237b* (family with sequence similarity 237, member B) that encodes an orexigenic hormone microprotein (Martinez et al. 2023). Methylation of *PCED1A* (PC-esterase domain containing 1 A) in human was affected by dietary fat quality (Voisin et al. 2015), and PCED1A might affect cellular rearrangement during adipocyte expansion in muscles (Silva-Vignato et al. 2022). Transcription factor ZFP536 (zinc finger protein 536) is involved in neurone differentiation (Qin et al. 2009) and was associated with both Attention-Deficit/Hyperactivity Disorder (ADHD) and excessive body weight (Dmitrzak-Weglarz et al. 2021), linking psychological disorders with obesity.

Other candidate genes include *4930595O18Rik*, *Angpt1*, *Atpsckmt*, *Cpped1*, *Erc2*, *Gm17131*, *Gm36633*, *Mast4*, *Prr5l*, *Tmem132d*, and *Trim24*. As for *4930441H08Rik*, no relevant information exists for *4930595O18Rik*. Interestingly though, as *4930441H08Rik*, it is predicted to target both *Hal* and not-yet mentioned *Fam98b* that is involved in liver fibrosis (Pazo et al. 2019), cancer (Akter et al. 2017), and activation of mRNA translation (Pazo et al. 2019). Among the top targets involved in obesity include *Pde6h* (phosphodiesterase 6 H, cGMP-specific, cone, gamma) (Sosa-Madrid et al. 2020), *Lrp5* (low density lipoprotein receptor-related protein 5) (Guo et al. 2006), potentially lncRNA *Gm37985* that is predicted to target *Hif3a* (hypoxia inducible factor 3, alpha subunit) (Mikec et al. 2022), and circRNA *Arf3* (ADP-ribosylation factor 3) (Rashad et al. 2023). Interestingly, as hypoxia is associated with obesity, angiogenesis marker ANGPT1 (angiopoietin 1) correlates positively with adipocyte size and body mass index-standard deviation score (BMI-SDS), a measure to define childhood obesity (Gaebler et al. 2019). *CPPED1* (calcineurin-like phosphoesterase domain containing 1) expression negatively affect glucose metabolism in human adipocytes (Vaittinen et al. 2013). ERC2 (ELKS/RAB6-interacting/CAST family member 2) is responsible for neurotransmitter release at inhibitory synapses (Kaeser et al. 2009) and was associated with body weight gain and feed conversion ratio in chicken as identified by the GWAS (Marchesi et al. 2021). The lncRNA *Gm36633* potentially also targets, among others, the above-mentioned *Fam98b* and *Igf2*, suggesting a coordinate role of the identified lncRNAs *4930595O18Rik*, *4930441H08Rik*, and *Gm36633* in obesity.

Moreover, *MAST4* (microtubule associated serine/threonine kinase family member 4), involved in neurodevelopment (Zhang et al. 2023), is repressed by glucocorticoids (Nguyen et al. 2022). In addition, a knock-down of numerous microtubule-binding or -associated proteins including *MAST4* caused a change in fat accumulation during adipogenesis (Söhle et al. 2012), and higher expression of *MAST4* was observed in the fat compared to the lean chicken line (Wang et al. 2021b). PRR5L (proline rich 5 like, also known as Protor-1 or 2) is part of the mTORC2 complex (Pearce et al. 2007), which is involved in various cellular processes including fatty acid and lipid synthesis (Guri et al. 2017). *PRR5L* expression was found differential in visceral adipose tissue of obesity patients (Guo et al. 2018) and found to be required for efficient mTORC2-mediated activation of SGK1 (serum/glucocorticoid regulated kinase 1) (Pearce et al. 2011). SGK1 is upregulated in obesity (Li et al. 2013; Bapat et al. 2022) and its genetic inhibition prevents obesity-related atrial fibrillation (Bapat et al. 2022). *TMEM132D* (transmembrane protein 132D) is primarily known for its association with addiction and anxiety (Hodgson et al. 2016). However, more recent studies also linked this gene with metabolic syndrome (Wan et al. 2021) and circadian rhythms (Li and Zhao 2020). Another interesting candidate is *Trim24* (tripartite motif-containing 24), an insulin dependent regulator of transcription and ubiquitination-dependent protein degradation (Wei et al. 2022). Wei et al. (2022) showed that TRIM24 interacts with components of processing bodies, which consequently stabilises *Pparγ* (peroxisome proliferator activated receptor gamma) mRNA (Wei et al. 2022), a master regulator of adipogenesis and obesity (Shao et al. 2016).

Taken together, a significant number of genes carrying potentially impactful variants were identified, including those previously related to obesity, as well as some novel potential candidates. In the present study, we primarily focused on SNPs with GERP score above 6 located in regulatory regions or having potential moderate or high impact on protein function, however, SNPs with lower GERP score and genes with other variant biotypes need to be explored in future studies.

## Conclusion

In conclusion, our sequencing and downstream bioinformatics analyses of two unique mouse models for polygenic obesity and healthy leanness revealed numerous genetic differences between the two lines and identified a plethora of genetic variants with potentially negative effects on gene function, including those previously associated with obesity. Given the growing catalogue of genetic polymorphisms in mice, this study provides a valuable resource of candidate genes for researchers to evaluate causality and function in obesity or leanness.

### Electronic supplementary material

Below is the link to the electronic supplementary material.

Supplementary Material 1

Supplementary Material 2

Supplementary Material 3

Supplementary Material 4

Supplementary Material 5

Supplementary Material 6

Supplementary Material 7

Supplementary Material 8

Supplementary Material 9

Supplementary Material 10

Supplementary Material 11

Supplementary Material 12

Supplementary Material 13

Supplementary Material 14

Supplementary Material 15

Supplementary Material 16

Supplementary Material 17

Supplementary Material 18

## Data Availability

The data are available on request.
