# Supplementary figures and images for: Whole genome sequencing of mouse lines divergently selected for fatness (FLI) and leanness (FHI) revealed several genetic variants as candidates for novel obesity genes
Source: Genes Genomics. 2024 Mar 14;46(5):557–75. doi: 10.1007/s13258-024-01507-9 (PMC11024027; doi:10.1007/s13258-024-01507-9)

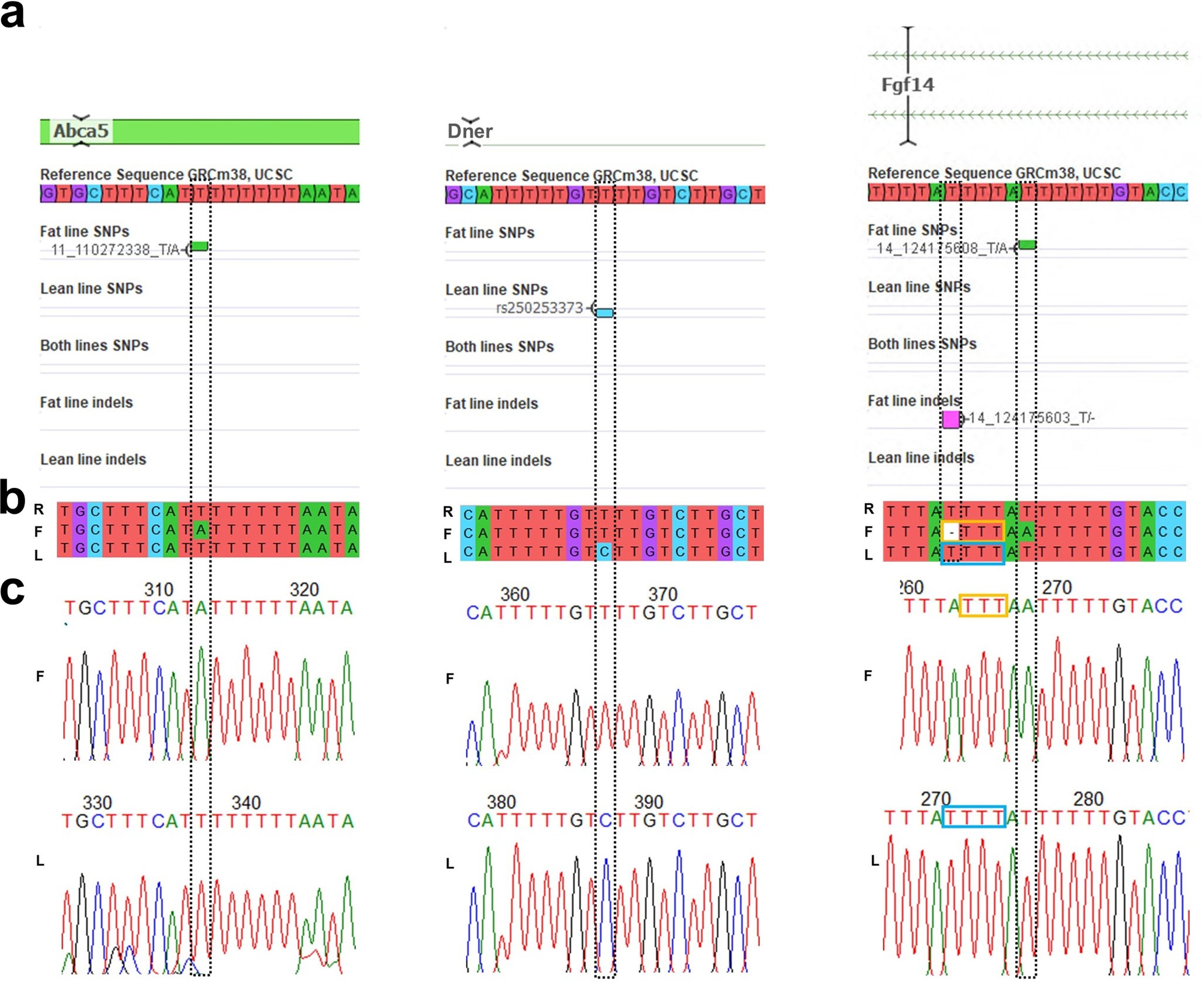

Supplement: Supplementary file 1 — Supplementary Material 1 [file 13258_2024_1507_MOESM1_ESM.jpg]

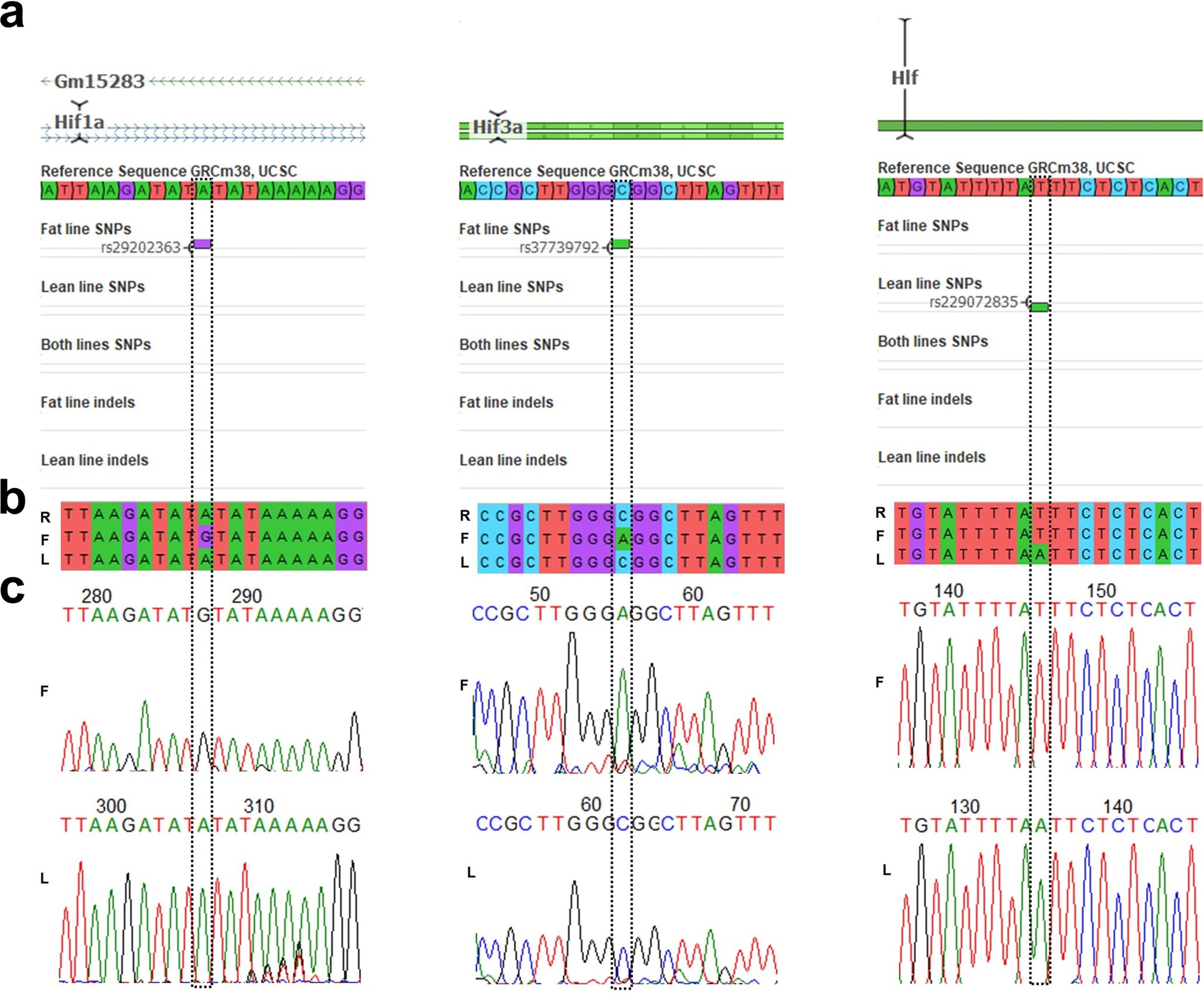

Supplement: Supplementary file 2 — Supplementary Material 2 [file 13258_2024_1507_MOESM2_ESM.jpg]

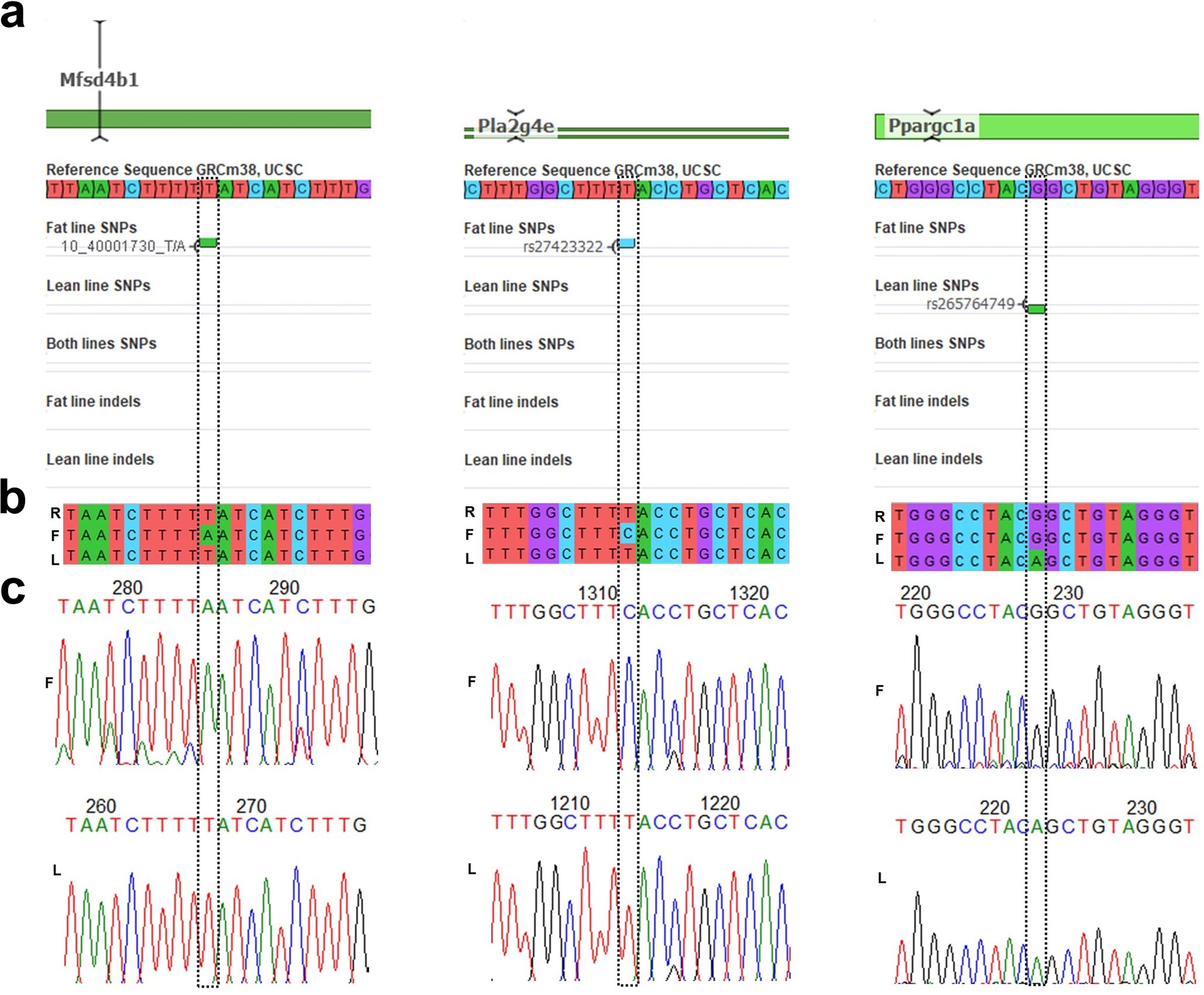

Supplement: Supplementary file 3 — Supplementary Material 3 [file 13258_2024_1507_MOESM3_ESM.jpg]

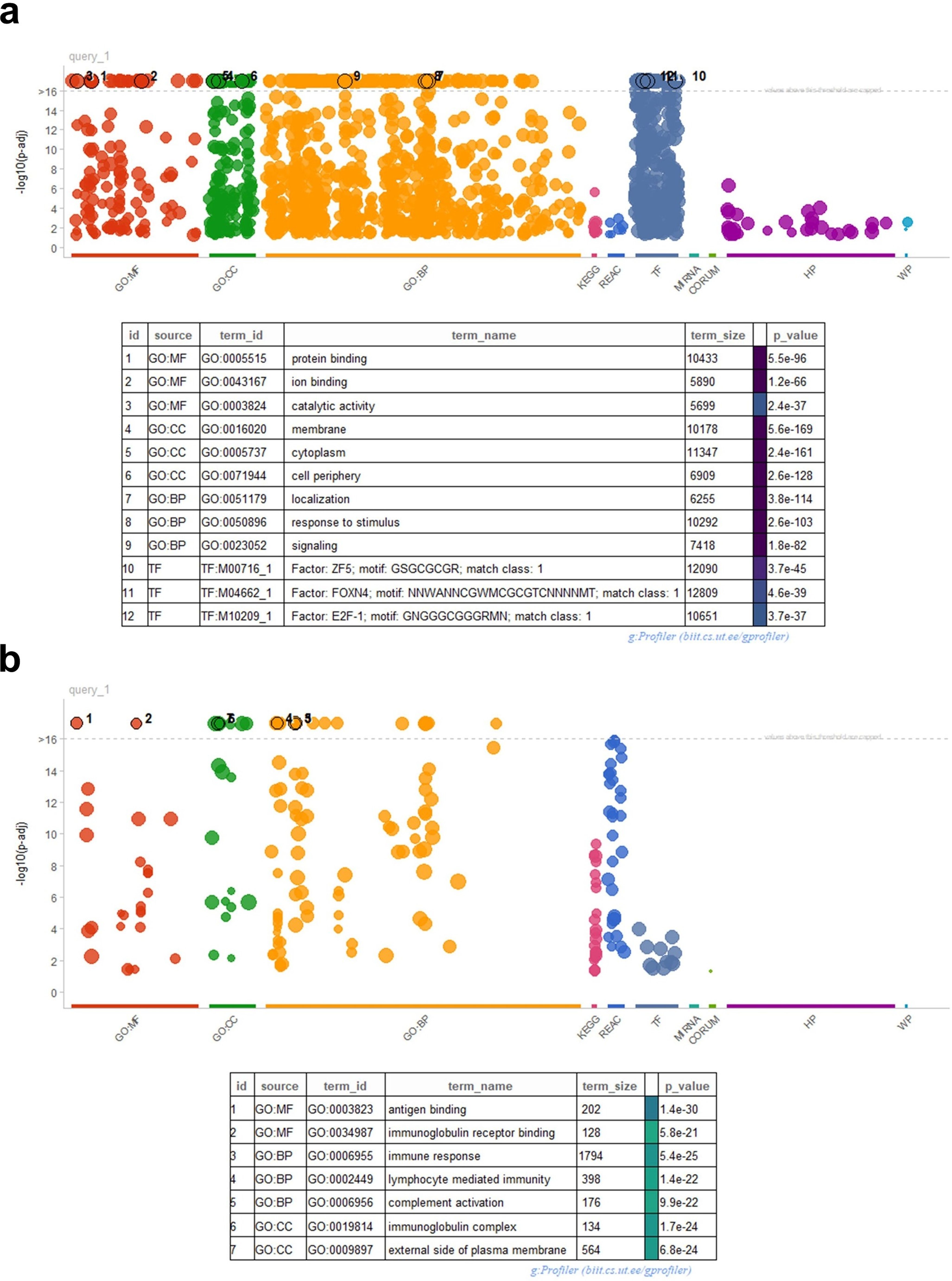

Supplement: Supplementary file 4 — Supplementary Material 4 [file 13258_2024_1507_MOESM4_ESM.jpg]

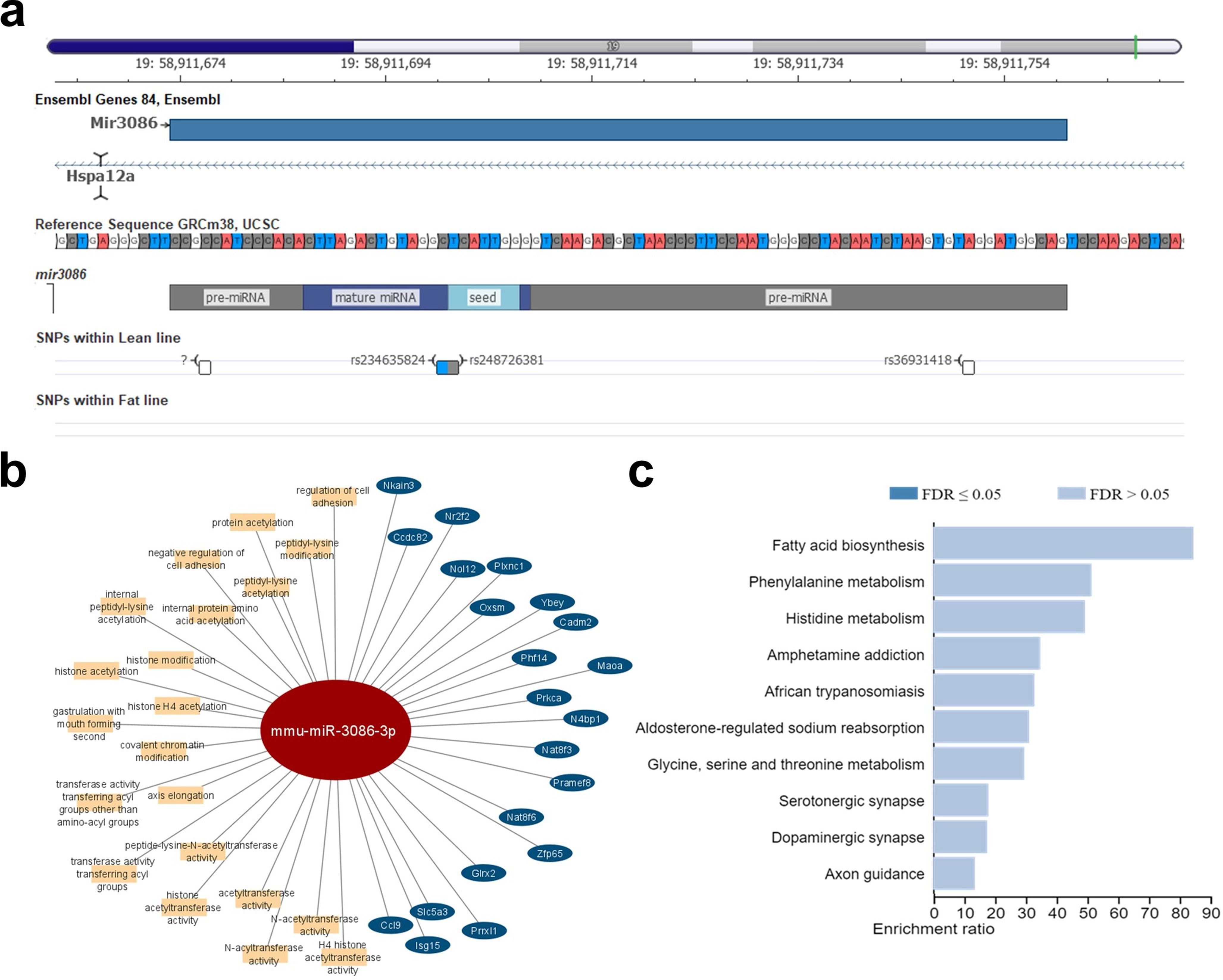

Supplement: Supplementary file 5 — Supplementary Material 5 [file 13258_2024_1507_MOESM5_ESM.jpg]

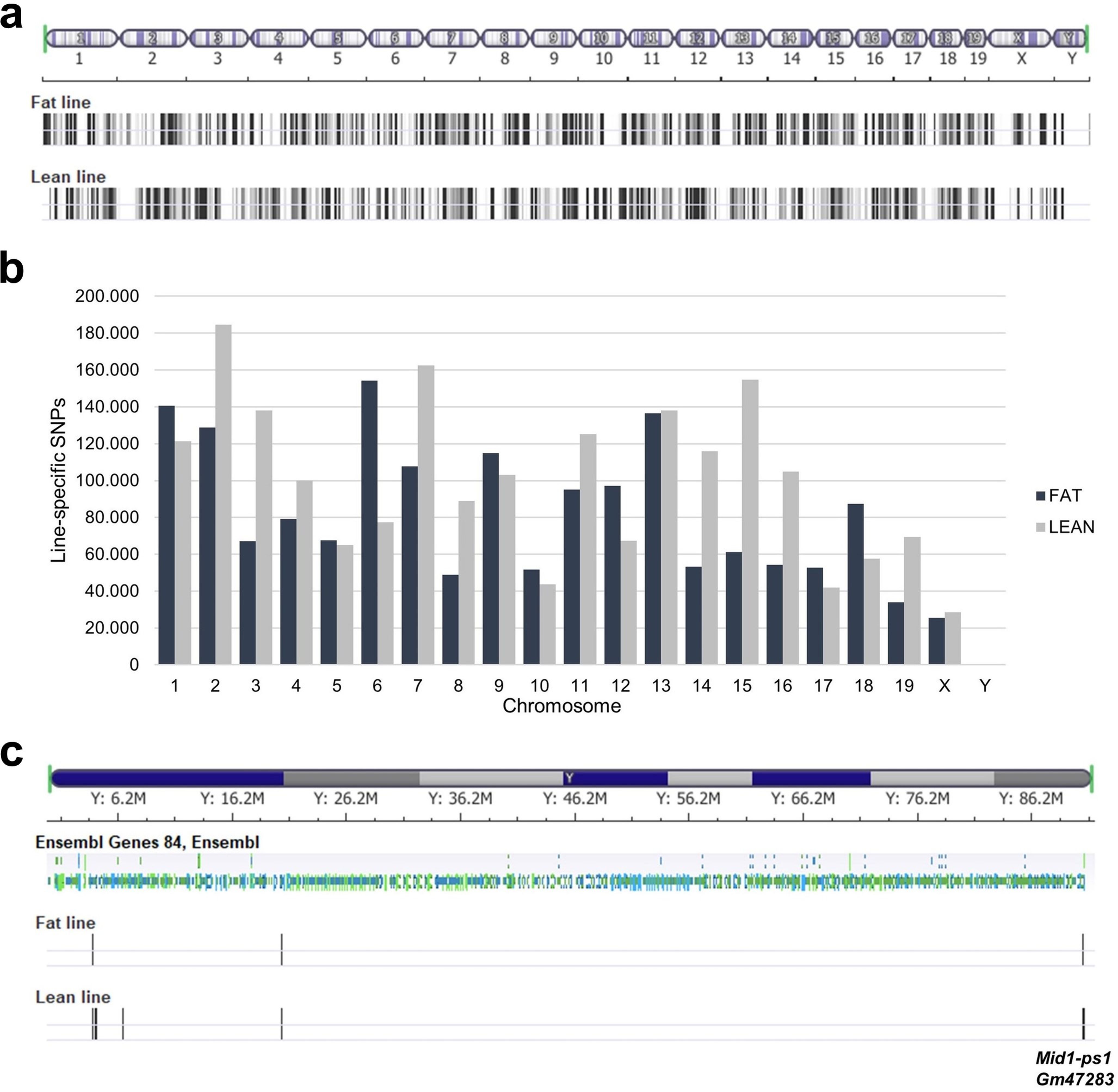

Supplement: Supplementary file 6 — Supplementary Material 6 [file 13258_2024_1507_MOESM6_ESM.jpg]

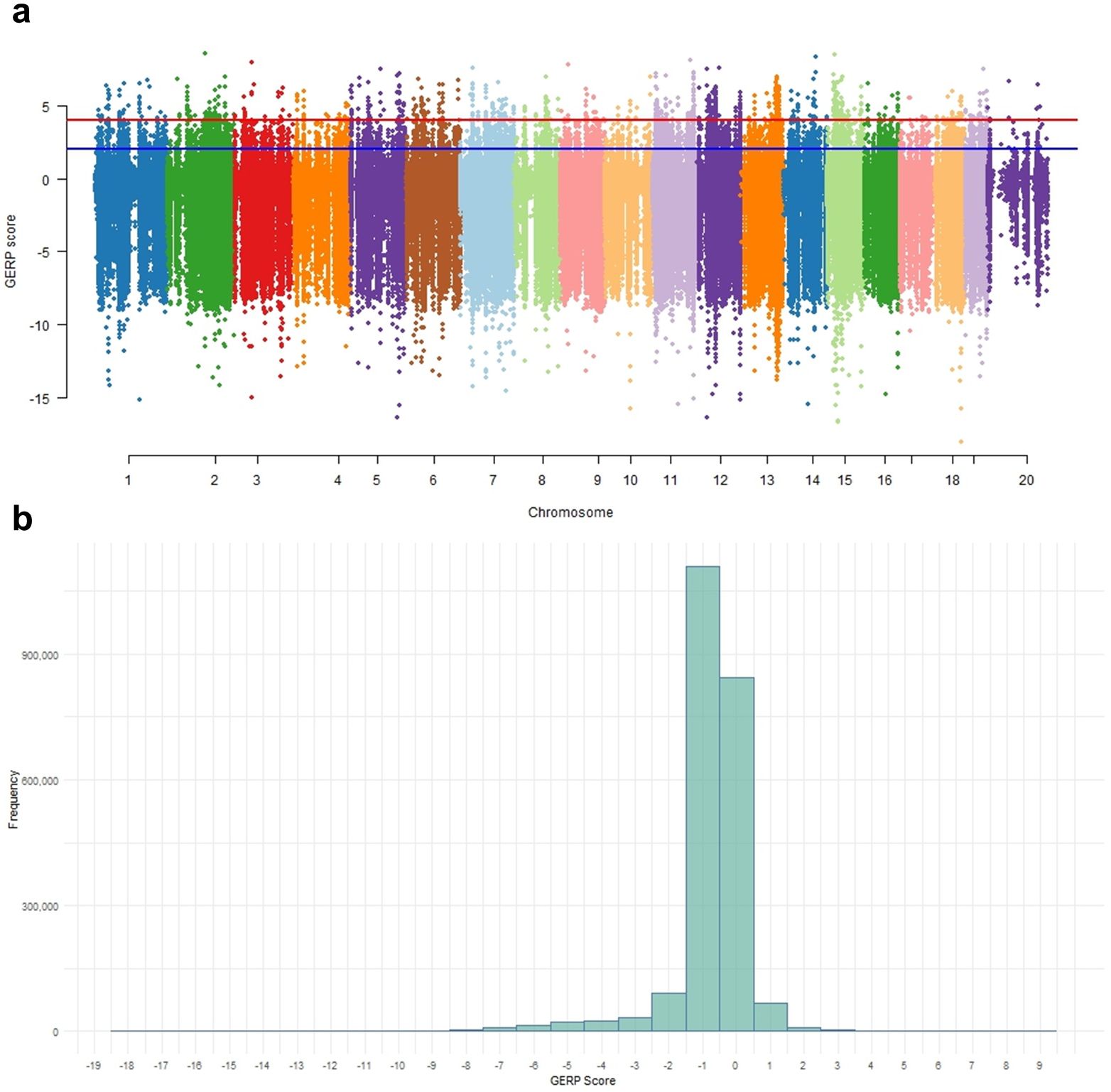

Supplement: Supplementary file 13 — Supplementary Material 13 [file 13258_2024_1507_MOESM13_ESM.jpg]

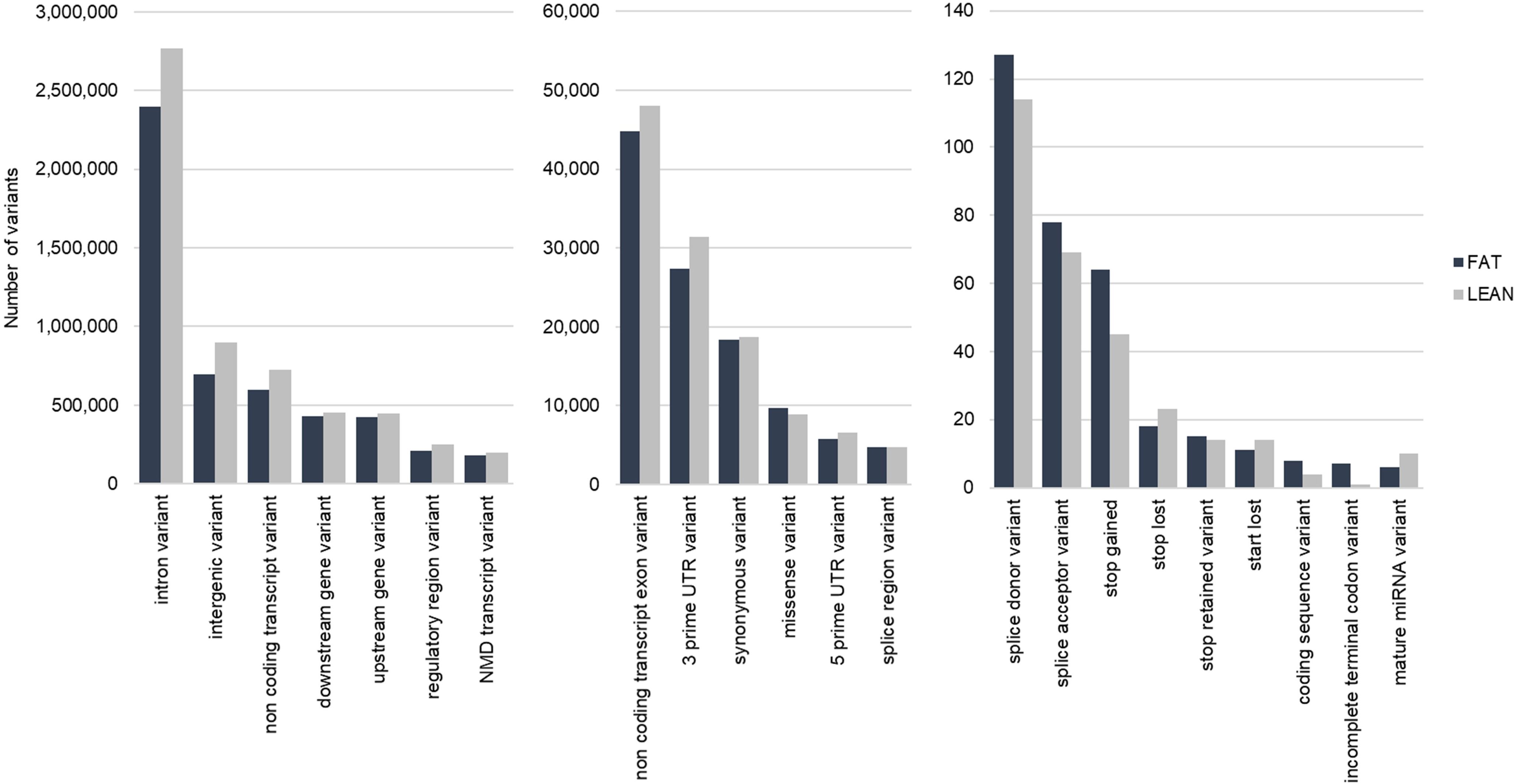

Supplement: Supplementary file 14 — Supplementary Material 14 [file 13258_2024_1507_MOESM14_ESM.jpg]

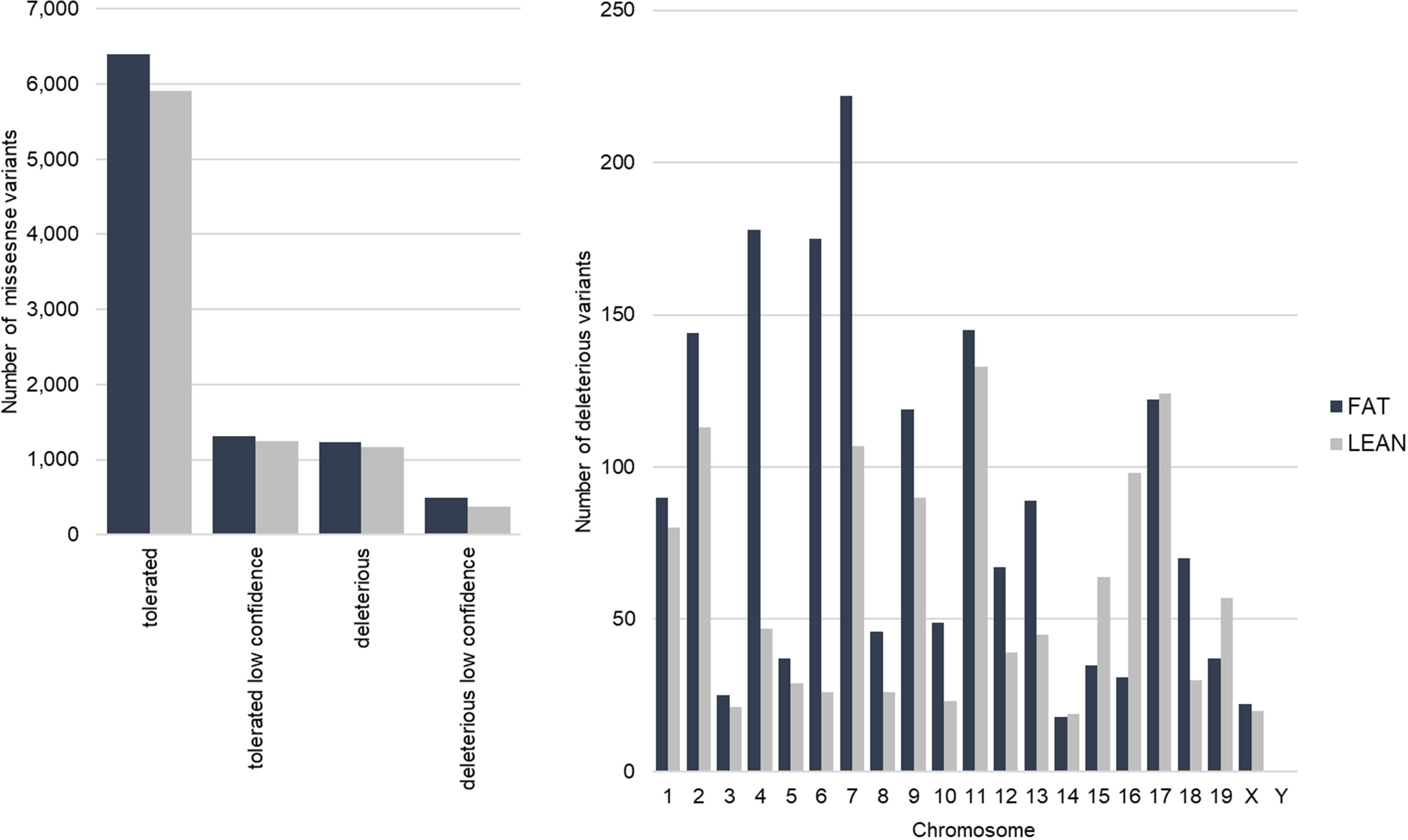

Supplement: Supplementary file 15 — Supplementary Material 15 [file 13258_2024_1507_MOESM15_ESM.jpg]

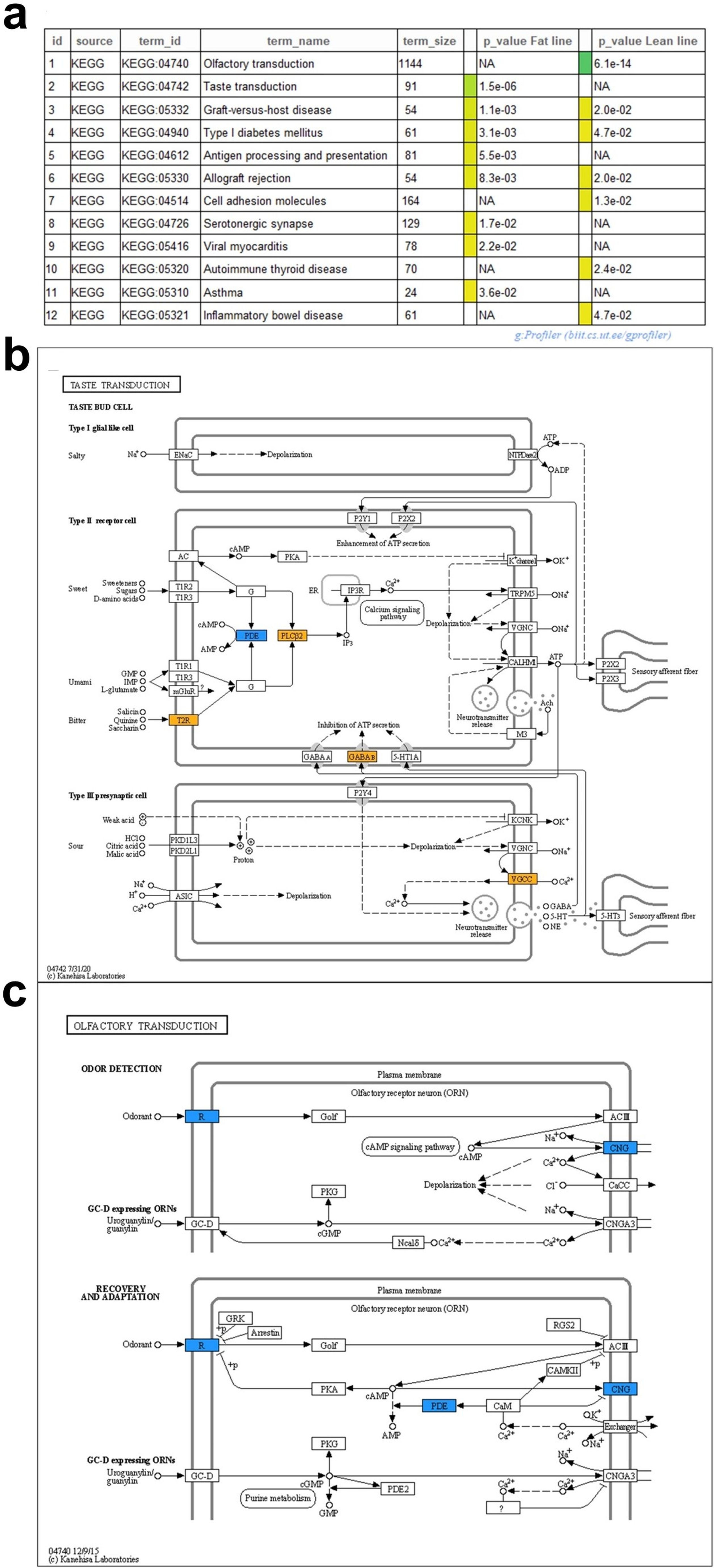

Supplement: Supplementary file 16 — Supplementary Material 16 [file 13258_2024_1507_MOESM16_ESM.jpg]

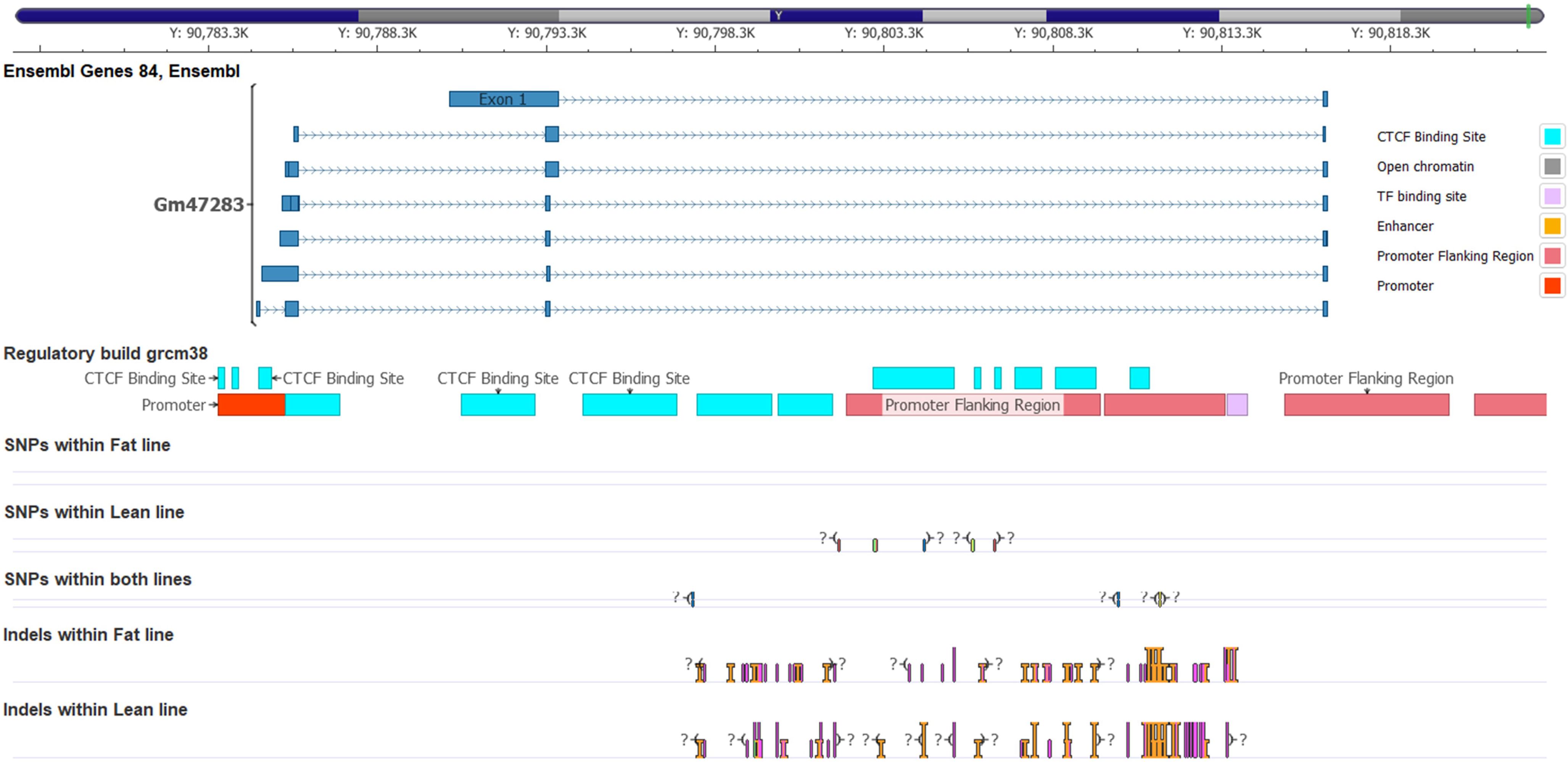

Supplement: Supplementary file 17 — Supplementary Material 17 [file 13258_2024_1507_MOESM17_ESM.jpg]
