# Supplementary material for: Whole genome sequencing of mouse lines divergently selected for fatness (FLI) and leanness (FHI) revealed several genetic variants as candidates for novel obesity genes
Source: Genes Genomics. 2024 Mar 14;46(5):557–75. doi: 10.1007/s13258-024-01507-9 (PMC11024027; doi:10.1007/s13258-024-01507-9)
Supplement: Supplementary file 7 — Supplementary Material 7 [file 13258_2024_1507_MOESM7_ESM.docx]

**Supplementary Table S2** Predicted consequences of novel SNPs identified in the Fat and Lean mouse lines.

| **Variant consequences** | **Impact** | **Fat** | **Lean** | **Both** | **SUM** |
| --- | --- | --- | --- | --- | --- |
| splice acceptor variant | high | 14 | 19 | 19 | 52 |
| splice donor variant | high | 20 | 20 | 28 | 68 |
| stop gained | high | 18 | 10 | 11 | 39 |
| stop lost | high | 1 | 4 | 6 | 11 |
| start lost | high | 1 | 3 | 6 | 10 |
| **SUM** | **high** | **54** | **56** | **70** | **180** |
| **missense variant** | **moderate** | **1162 (296 dt*)** | **957 (229 dt*)** | **1458 (254 dt*)** | **3577 (779 dt*)** |
| splice region variant | low | 370 | 336 | 476 | 1182 |
| incomplete terminal codon variant | low | 3 | 0 | 3 | 6 |
| stop retained variant | low | 0 | 4 | 2 | 6 |
| synonymous variant | low | 1472 | 1345 | 1922 | 4739 |
| **SUM** | **low** | **1845** | **1685** | **2403** | **5933** |
| mature miRNA variant | modified | 1 | 0 | 4 | 5 |
| 5 prime UTR variant | modified | 1097 | 1288 | 1610 | 3995 |
| 3 prime UTR variant | modified | 3652 | 5112 | 5454 | 14218 |
| non-coding transcript exon variant | modified | 8095 | 8230 | 10407 | 26732 |
| intron variant | modified | 155974 | 169146 | 198657 | 523777 |
| upstream gene variant | modified | 52340 | 55469 | 74808 | 182617 |
| downstream gene variant | modified | 53249 | 58027 | 78638 | 189914 |
| regulatory region variant | modified | 36248 | 41183 | 53070 | 130501 |
| intergenic variant | modified | 164798 | 212380 | 207718 | 584896 |
| **SUM** | **modified** | **475454** | **550835** | **630366** | **1656655** |
| * - dt: deleterious | | | | | |
