# Supplementary material for: Whole genome sequencing of mouse lines divergently selected for fatness (FLI) and leanness (FHI) revealed several genetic variants as candidates for novel obesity genes
Source: Genes Genomics. 2024 Mar 14;46(5):557–75. doi: 10.1007/s13258-024-01507-9 (PMC11024027; doi:10.1007/s13258-024-01507-9)
Supplement: Supplementary file 8 — Supplementary Material 8 [file 13258_2024_1507_MOESM8_ESM.docx]

**Supplementary Table S3** Genes with at least 3000 line-specific SNPs identified in the Lean and Fat lines.

| **Gene ID** | **Symbol** | **Gene name** | **Feature Type** | **Count** | **Line** |
| --- | --- | --- | --- | --- | --- |
| ENSMUSG00000022311 | *Csmd3* | CUB and Sushi multiple domains 3 | protein coding gene | 10509 | Lean |
| ENSMUSG00000062209 | *Erbb4* | erb-b2 receptor tyrosine kinase 4 | protein coding gene | 8019 | Lean |
| ENSMUSG00000078598 | *Skint5* | selection and upkeep of intraepithelial T cells 5 | protein coding gene | 7449 | Fat |
| ENSMUSG00000037940 | *Inpp4b* | inositol polyphosphate-4-phosphatase, type II | protein coding gene | 7050 | Lean |
| ENSMUSG00000029763 | *Exoc4* | exocyst complex component 4 | protein coding gene | 6557 | Fat |
| ENSMUSG00000092329 | *Galnt2l* | polypeptide N-acetylgalactosaminyltransferase 2-like | protein coding gene | 6315 | Fat |
| ENSMUSG00000055026 | *Gabrg3* | gamma-aminobutyric acid (GABA) A receptor, subunit gamma 3 | protein coding gene | 5754 | Lean |
| ENSMUSG00000068205 | *Macrod2* | mono-ADP ribosylhydrolase 2 | protein coding gene | 5620 | Fat |
| ENSMUSG00000027737 | *Slc7a11* | solute carrier family 7 (cationic amino acid transporter, y+ system), member 11 | protein coding gene | 5456 | Lean |
| ENSMUSG00000006191 | *Cdkal1* | CDK5 regulatory subunit associated protein 1-like 1 | protein coding gene | 5318 | Lean |
| ENSMUSG00000048078 | *Tenm4* | teneurin transmembrane protein 4 | protein coding gene | 5280 | Lean |
| ENSMUSG00000025909 | *Sntg1* | syntrophin, gamma 1 | protein coding gene | 4919 | Fat |
| ENSMUSG00000054843 | *Atrnl1* | attractin like 1 | protein coding gene | 4918 | Lean |
| ENSMUSG00000031129 | *Slc9a9* | solute carrier family 9 (sodium/hydrogen exchanger), member 9 | protein coding gene | 4901 | Fat |
| ENSMUSG00000060843 | *Ctnna3* | catenin (cadherin associated protein), alpha 3 | protein coding gene | 4870 | Fat |
| ENSMUSG00000106379 | *Lhfpl3* | lipoma HMGIC fusion partner-like 3 | protein coding gene | 4629 | Fat |
| ENSMUSG00000055067 | *Smyd3* | SET and MYND domain containing 3 | protein coding gene | 4577 | Fat |
| ENSMUSG00000041540 | *Sox5* | SRY (sex determining region Y)-box 5 | protein coding gene | 4395 | Fat |
| ENSMUSG00000034488 | *Edil3* | EGF-like repeats and discoidin I-like domains 3 | protein coding gene | 4391 | Lean |
| ENSMUSG00000023826 | *Prkn* | parkin RBR E3 ubiquitin protein ligase | protein coding gene | 4388 | Lean |
| ENSMUSG00000087194 | *Skint6* | selection and upkeep of intraepithelial T cells 6 | protein coding gene | 4278 | Fat |
| ENSMUSG00000056899 | *Immp2l* | IMP2 inner mitochondrial membrane peptidase-like *(S. cerevisiae*) | protein coding gene | 4226 | Lean |
| ENSMUSG00000024552 | *Slc14a2* | solute carrier family 14 (urea transporter), member 2 | protein coding gene | 4221 | Fat |
| ENSMUSG00000033676 | *Gabrb3* | gamma-aminobutyric acid (GABA) A receptor, subunit beta 3 | protein coding gene | 4114 | Lean |
| ENSMUSG00000022021 | *Diaph3* | diaphanous related formin 3 | protein coding gene | 4106 | Lean |
| ENSMUSG00000068205 | *Macrod2* | mono-ADP ribosylhydrolase 2 | protein coding gene | 4090 | Lean |
| ENSMUSG00000027971 | *Ndst4* | N-deacetylase/N-sulfotransferase (heparin glucosaminyl) 4 | protein coding gene | 3931 | Fat |
| ENSMUSG00000025551 | *Fgf14* | fibroblast growth factor 14 | protein coding gene | 3854 | Fat |
| ENSMUSG00000049336 | *Tenm2* | teneurin transmembrane protein 2 | protein coding gene | 3812 | Lean |
| ENSMUSG00000032985 | *5730522E02Rik* | RIKEN cDNA 5730522E02 gene | protein coding gene | 3811 | Lean |
| ENSMUSG00000021536 | *Adcy2* | adenylate cyclase 2 | protein coding gene | 3648 | Fat |
| ENSMUSG00000049336 | *Tenm2* | teneurin transmembrane protein 2 | protein coding gene | 3535 | Fat |
| ENSMUSG00000024713 | *Pcsk5* | proprotein convertase subtilisin/kexin type 5 | protein coding gene | 3497 | Lean |
| ENSMUSG00000021596 | *Mctp1* | multiple C2 domains, transmembrane 1 | protein coding gene | 3491 | Fat |
| ENSMUSG00000073565 | *Prr16* | proline rich 16 | protein coding gene | 3455 | Lean |
| ENSMUSG00000034751 | *Mast4* | microtubule associated serine/threonine kinase family member 4 | protein coding gene | 3393 | Fat |
| ENSMUSG00000052387 | *Trpm3* | transient receptor potential cation channel, subfamily M, member 3 | protein coding gene | 3335 | Fat |
| ENSMUSG00000078591 | *Hs3st4* | heparan sulfate (glucosamine) 3-O-sulfotransferase 4 | protein coding gene | 3307 | Lean |
| ENSMUSG00000039007 | *Cpq* | carboxypeptidase Q | protein coding gene | 3225 | Fat |
| ENSMUSG00000043456 | *Zfp536* | zinc finger protein 536 | protein coding gene | 3209 | Lean |
| ENSMUSG00000004698 | *Hdac9* | histone deacetylase 9 | protein coding gene | 3204 | Fat |
| ENSMUSG00000060534 | *Dcc* | deleted in colorectal carcinoma | protein coding gene | 3185 | Fat |
| ENSMUSG00000039913 | *Pak5* | p21 (RAC1) activated kinase 5 | protein coding gene | 3183 | Lean |
| ENSMUSG00000103458 | *Gm37013* | predicted gene, 37013 | protein coding gene | 3055 | Fat |
| ENSMUSG00000030450 | *Oca2* | oculocutaneous albinism II | protein coding gene | 3039 | Lean |
| ENSMUSG00000021730 | *Hcn1* | hyperpolarization activated cyclic nucleotide gated potassium channel 1 | protein coding gene | 3031 | Lean |
| ENSMUSG00000034098 | *Fstl5* | follistatin-like 5 | protein coding gene | 3013 | Lean |
| ENSMUSG00000038372 | *Gmds* | GDP-mannose 4, 6-dehydratase | protein coding gene | 3012 | Lean |
