# Supplementary material for: Whole genome sequencing of mouse lines divergently selected for fatness (FLI) and leanness (FHI) revealed several genetic variants as candidates for novel obesity genes
Source: Genes Genomics. 2024 Mar 14;46(5):557–75. doi: 10.1007/s13258-024-01507-9 (PMC11024027; doi:10.1007/s13258-024-01507-9)
Supplement: Supplementary file 9 — Supplementary Material 9 [file 13258_2024_1507_MOESM9_ESM.docx]

**Supplementary Table S4** Genes with at least four line-specific SNPs per 100 bp identified in the Lean and Fat lines.

| Gene ID | Symbol | Name | Feature Type | SNP count | Gene width [bp] | SNP/100 bp | Line |
| --- | --- | --- | --- | --- | --- | --- | --- |
| ENSMUSG00000064386 | *Gm26449* | predicted gene, 26449 | snRNA gene | 12 | 104 | 11.5 | Fat |
| ENSMUSG00000104931 | *Igkv1-136* | immunoglobulin kappa chain variable 1-136 | pseudogenic gene segment | 25 | 297 | 8.4 | Fat |
| ENSMUSG00000065702 | *Gm22828* | predicted gene, 22828 | snRNA gene | 7 | 107 | 6.5 | Fat |
| ENSMUSG00000090109 | *Ear-ps10* | eosinophil-associated, ribonuclease A family, pseudogene 10 | pseudogene | 29 | 460 | 6.3 | Lean |
| ENSMUSG00000107784 | *Gm43943* | predicted gene, 43943 | pseudogene | 7 | 113 | 6.2 | Fat |
| ENSMUSG00000118617 | *AC152418.1* | aurora kinase C (Aurkc) pseudogene | pseudogene | 5 | 84 | 6.0 | Fat |
| ENSMUSG00000096250 | *Ighd2-6* | immunoglobulin heavy diversity 2-6 | gene segment | 1 | 17 | 5.9 | Fat |
| ENSMUSG00000098486 | *Gm27385* | predicted gene, 27385 | snRNA gene | 6 | 104 | 5.8 | Fat |
| ENSMUSG00000102213 | *Gm37489* | predicted gene, 37489 | pseudogene | 9 | 159 | 5.7 | Fat |
| ENSMUSG00000064905 | *Gm25403* | predicted gene, 25403 | misc RNA | 8 | 143 | 5.6 | Fat |
| ENSMUSG00000065813 | *n-R5s3* | nuclear encoded rRNA 5S 3 | rRNA | 6 | 112 | 5.4 | Lean |
| ENSMUSG00000065018 | *Gm23511* | predicted gene, 23511 | snRNA | 8 | 164 | 4.9 | Lean |
| ENSMUSG00000115282 | *Gm49055* | predicted gene, 49055 | pseudogene | 18 | 370 | 4.9 | Lean |
| ENSMUSG00000077155 | *Gm24582* | predicted gene, 24582 | snRNA gene | 5 | 103 | 4.9 | Lean |
| ENSMUSG00000095826 | *Gm9002* | predicted gene 9002 | pseudogene | 24 | 499 | 4.8 | Fat |
| ENSMUSG00000088854 | *Gm24725* | predicted gene, 24725 | snRNA gene | 6 | 125 | 4.8 | Fat |
| ENSMUSG00000099140 | *Mir7237* | microRNA 7237 | miRNA gene | 3 | 63 | 4.8 | Fat |
| ENSMUSG00000106222 | *Rprl1* | ribonuclease P RNA-like 1 | RNase P RNA gene | 11 | 238 | 4.6 | Fat |
| ENSMUSG00000077298 | *Gm24127* | predicted gene, 24127 | snoRNA gene | 6 | 130 | 4.6 | Lean |
| ENSMUSG00000092813 | *Mir3086* | microRNA 3086 | miRNA gene | 4 | 87 | 4.6 | Lean |
| ENSMUSG00000088090 | *Gm23668* | predicted gene, 23668 | rRNA | 5 | 111 | 4.5 | Fat |
| ENSMUSG00000076305 | *Gm23063* | predicted gene, 23063 | miRNA gene | 4 | 93 | 4.3 | Lean |
| ENSMUSG00000105451 | *Gm43220* | predicted gene 43220 | pseudogenic gene segment | 12 | 282 | 4.3 | Fat |
| ENSMUSG00000066457 | *S100a11-ps* | S100 calcium binding protein A11, pseudogene | pseudogene | 13 | 306 | 4.2 | Fat |
| ENSMUSG00000105163 | *Gm4873* | predicted gene 4873 | pseudogene | 51 | 1203 | 4.2 | Fat |
| ENSMUSG00000114063 | *Gm47555* | predicted gene, 47555 | pseudogene | 14 | 336 | 4.2 | Lean |
| ENSMUSG00000083967 | *Gm15115* | predicted gene 15115 | pseudogene | 11 | 264 | 4.2 | Lean |
| ENSMUSG00000111543 | *Olfr1139-ps1* | olfactory receptor 1139, pseudogene 1 | pseudogene | 7 | 168 | 4.2 | Lean |
| ENSMUSG00000117348 | *Gm34658* | predicted gene, 34658 | pseudogene | 15 | 362 | 4.1 | Fat |
| ENSMUSG00000077317 | *Gm25785* | predicted gene, 25785 | snRNA gene | 5 | 125 | 4.0 | Lean |
