# Supplementary material for: Whole genome sequencing of mouse lines divergently selected for fatness (FLI) and leanness (FHI) revealed several genetic variants as candidates for novel obesity genes
Source: Genes Genomics. 2024 Mar 14;46(5):557–75. doi: 10.1007/s13258-024-01507-9 (PMC11024027; doi:10.1007/s13258-024-01507-9)
Supplement: Supplementary file 10 — Supplementary Material 10 [file 13258_2024_1507_MOESM10_ESM.docx]

**Supplementary Table S5** Obesity-related genes (IMPC) with line-specific and shared predicted deleterious missense variants in the Fat and Lean line.

| **FAT LINE** | | | **LEAN LINE** | | | **BOTH LINES** | | |
| --- | --- | --- | --- | --- | --- | --- | --- | --- |
| **Symbol** | **Name** | **All and novel (x/x) DMVs^*^** | **Symbol** | **Name** | **All and novel (x/x) DMVs^*^** | **Symbol** | **Name** | **All and novel (x/x) DMVs^*^** |
| *2210408I21Rik* | RIKEN cDNA 2210408I21 gene | 1/0 | *Alg8* | asparagine-linked glycosylation 8 (alpha-1,3-glucosyltransferase) | 1/0 | *2210408I21Rik* | RIKEN cDNA 2210408I21 gene | 1/0 |
| *Agbl1* | ATP/GTP binding protein-like 1 | 1/1 | *Alpk2* | alpha-kinase 2 | 1/0 | *Akap9* | A kinase (PRKA) anchor protein (yotiao) 9 | 1/0 |
| *Cep250* | centrosomal protein 250 | 4/0 | *Aspm* | abnormal spindle microtubule assembly | 1/0 | *Anxa6* | annexin A6 | 1/0 |
| *Fam81b* | family with sequence similarity 81, member B | 1/0 | *Chsy3* | chondroitin sulfate synthase 3 | 1/1 | *Cdh23* | cadherin 23 (otocadherin) | 1/0 |
| *Il6st* | interleukin 6 signal transducer | 1/0 | *Csmd3* | CUB and Sushi multiple domains 3 | 2/1 | *Cidec* | cell death-inducing DFFA-like effector c | 1/0 |
| *Mamld1* | mastermind-like domain containing 1 | 1/0 | *D430041D05Rik* | RIKEN cDNA D430041D05 gene | 5/0 | *E4f1* | E4F transcription factor 1 | 1/0 |
| *Nbas* | neuroblastoma amplified sequence | 2/1 | *D630045J12Rik* | RIKEN cDNA D630045J12 gene | 4/0 | *Epx* | eosinophil peroxidase | 1/1 |
| *Pth1r* | parathyroid hormone 1 receptor | 1/0 | *Dock9* | dedicator of cytokinesis 9 | 1/0 | *Frmd5* | FERM domain containing 5 | 3/0 |
| *Sema4d* | sema domain, immunoglobulin domain (Ig), transmembrane domain (TM) and short cytoplasmic domain, (semaphorin) 4D | 1/0 | *Gpr15* | G protein-coupled receptor 15 | 1/0 | *Hook3* | hook microtubule tethering protein 3 | 1/0 |
| *Slco1b2* | solute carrier organic anion transporter family, member 1b2 | 1/0 | *Kbtbd8* | kelch repeat and BTB (POZ) domain containing 8 | 1/1 | *Hspbap1* | Hspb associated protein 1 | 1/1 |
| *Tulp3* | tubby-like protein 3 | 1/1 | *Phldb1* | pleckstrin homology like domain, family B, member 1 | 1/0 | *Il31ra* | interleukin 31 receptor A | 1/0 |
|  |  |  | *Zfp462* | zinc finger protein 462 | 1/0 | *Irak2* | interleukin-1 receptor-associated kinase 2 | 1/0 |
|  |  |  |  |  |  | *Jmjd4* | jumonji domain containing 4 | 1/0 |
|  |  |  |  |  |  | *Kmt2e* | lysine (K)-specific methyltransferase 2E | 1/0 |
|  |  |  |  |  |  | *Mep1a* | meprin 1 alpha | 2/0 |
|  |  |  |  |  |  | *Phkb* | phosphorylase kinase beta | 1/0 |
|  |  |  |  |  |  | *Rnase10* | ribonuclease, RNase A family, 10 (non-active) | 1/0 |
|  |  |  |  |  |  | *Sec31b* | Sec31 homolog B (S. cerevisiae) | 2/0 |
|  |  |  |  |  |  | *Slc37a1* | solute carrier family 37 (glycerol-3-phosphate transporter), member 1 | 2/0 |
|  |  |  |  |  |  | *Slc51b* | solute carrier family 51, beta subunit | 1/0 |
|  |  |  |  |  |  | *Stard3nl* | STARD3 N-terminal like | 3/0 |
|  |  |  |  |  |  | *Syce2* | synaptonemal complex central element protein 2 | 1/1 |
|  |  |  |  |  |  | *Tmem260* | transmembrane protein 260 | 1/0 |
|  |  |  |  |  |  | *Zbtb48* | zinc finger and BTB domain containing 48 | 1/0 |
| Obesity-related genes were obtained from the International Mouse Phenotyping Consortium (IMPC) database  * - DMVs: predicted deleterious missense variants | | |  |  |  |  |  |  |
