# Supplementary material for: Whole genome sequencing of mouse lines divergently selected for fatness (FLI) and leanness (FHI) revealed several genetic variants as candidates for novel obesity genes
Source: Genes Genomics. 2024 Mar 14;46(5):557–75. doi: 10.1007/s13258-024-01507-9 (PMC11024027; doi:10.1007/s13258-024-01507-9)
Supplement: Supplementary file 11 — Supplementary Material 11 [file 13258_2024_1507_MOESM11_ESM.docx]

**Supplementary Table S6** Protein-coding transcripts with the highest density of line-specific missense variants identified in the Fat and Lean mouse selection lines.

| **Transcript ID** | **Gene symbol** | **Gene name** | **Count** | **Protein length** | **Density** | **Line** |
| --- | --- | --- | --- | --- | --- | --- |
| ENSMUST00000106866 | *Hbb-bh2* | hemoglobin beta, bh2 | 10 | 147 | 14,7 | Lean |
| ENSMUST00000211947 | *Cx3cl1* | C-X3-C motif chemokine ligand 1 | 3 | 45 | 15,0 | Fat |
| ENSMUST00000205641 | *Hamp2* | hepcidin antimicrobial peptide 2 | 6 | 103 | 17,2 | Fat |
| ENSMUST00000194796 | *Kcnmb2* | potassium large conductance calcium-activated channel, subfamily M, beta member 2 | 2 | 36 | 18,0 | Lean |
| ENSMUST00000108514 | *Nlrp1b* | NLR family, pyrin domain containing 1B | 65 | 1177 | 18,1 | Fat |
| ENSMUST00000108515 | *Nlrp1b* | NLR family, pyrin domain containing 1B | 65 | 1177 | 18,1 | Fat |
| ENSMUST00000066719 | *Ang5* | angiogenin, ribonuclease A family, member 5 | 8 | 145 | 18,1 | Lean |
| ENSMUST00000065532 | *Tas2r136* | taste receptor, type 2, member 136 | 18 | 327 | 18,2 | Fat |
| ENSMUST00000099846 | *Or5w17* | olfactory receptor family 5 subfamily W member 17, pseudogene 1 | 17 | 311 | 18,3 | Lean |
| ENSMUST00000109753 | *Hamp2* | hepcidin antimicrobial peptide 2 | 20 | 367 | 18,4 | Lean |
| ENSMUST00000188157 | *Cd22* | CD22 antigen | 8 | 147 | 18,4 | Fat |
| ENSMUST00000081184 | *Or5p58* | olfactory receptor family 5 subfamily P member 58 | 17 | 323 | 19,0 | Fat |
| ENSMUST00000217304 | *Or5p58* | olfactory receptor family 5 subfamily P member 58 | 17 | 323 | 19,0 | Fat |
| ENSMUST00000189962 | *Krtdap* | keratinocyte differentiation associated protein | 3 | 57 | 19,0 | Fat |
| ENSMUST00000120115 | *Gm5678* (*Cbr1b*) | carbonyl reductase 1B | 14 | 277 | 19,8 | Lean |
| ENSMUST00000075329 | *Or10x4* | olfactory receptor family 10 subfamily X member 4 | 15 | 309 | 20,6 | Fat |
| ENSMUST00000032317 | *Tas2r103* | taste receptor, type 2, member 103 | 15 | 312 | 20,8 | Fat |
| ENSMUST00000174796 | *Fkbpl* | FK506 binding protein-like | 1 | 21 | 21,0 | Fat |
| ENSMUST00000099885 | *Or8k28* | olfactory receptor family 8 subfamily K member 28 | 14 | 313 | 22,4 | Lean |
| ENSMUST00000113071 | *Tmsb15b1* | thymosin beta 15b1 | 2 | 45 | 22,5 | Fat |
| ENSMUST00000067539 | *Tas2r109* | taste receptor, type 2, member 109 | 14 | 316 | 22,6 | Fat |
| ENSMUST00000221266 | *Gm10037* (*Krbox5*) | KRAB box domain containing 5 | 4 | 91 | 22,8 | Fat |
| ENSMUST00000221620 | *Gm10037* (*Krbox5*) | KRAB box domain containing 5 | 4 | 91 | 22,8 | Fat |
| ENSMUST00000043836 | *Macrod2* | mono-ADP ribosylhydrolase 2 | 5 | 114 | 22,8 | Fat |
| ENSMUST00000171980 | *Gm17631* | predicted gene, 17631 | 1 | 23 | 23,0 | Fat |
| ENSMUST00000040655 | *H2-Aa* | histocompatibility 2, class II antigen A, alpha | 11 | 256 | 23,3 | Lean |
| ENSMUST00000233343 | *Emilin2* | elastin microfibril interfacer 2 | 4 | 95 | 23,8 | Fat |
| ENSMUST00000082085 | *Tas2r131* | taste receptor, type 2, member 131 | 13 | 310 | 23,8 | Fat |
| ENSMUST00000073532 | *Gm13089* (*Pramel23*) | PRAME like 23 | 19 | 455 | 23,9 | Lean |
| ENSMUST00000237342 | *H2-Ab1* | histocompatibility 2, class II antigen A, beta 1 | 8 | 192 | 24,0 | Fat |
| ENSMUST00000164250 | *Zfp426* | Olfr1141 | 1 | 24 | 24,0 | Lean |
| ENSMUST00000162158 | *Skint1* | selection and upkeep of intraepithelial T cells 1 | 15 | 364 | 24,3 | Fat |
| ENSMUST00000106572 | *Skint8* | selection and upkeep of intraepithelial T cells 8 | 13 | 318 | 24,5 | Fat |
| ENSMUST00000233728 | *Emilin2* | elastin microfibril interfacer 2 | 4 | 98 | 24,5 | Fat |
| ENSMUST00000190753 | *Cd22* | CD22 antigen | 2 | 49 | 24,5 | Fat |
| ENSMUST00000222626 | *Gm10037* (*Krbox5*) | KRAB box domain containing 5 | 3 | 74 | 24,7 | Fat |
| ENSMUST00000171627 | *Skint5* | selection and upkeep of intraepithelial T cells 5 | 21 | 519 | 24,7 | Fat |
| ENSMUST00000177224 | *Mok* | MOK protein kinase | 4 | 99 | 24,8 | Fat |
