# Supplementary material for: Whole genome sequencing of mouse lines divergently selected for fatness (FLI) and leanness (FHI) revealed several genetic variants as candidates for novel obesity genes
Source: Genes Genomics. 2024 Mar 14;46(5):557–75. doi: 10.1007/s13258-024-01507-9 (PMC11024027; doi:10.1007/s13258-024-01507-9)
Supplement: Supplementary file 12 — Supplementary Material 12 [file 13258_2024_1507_MOESM12_ESM.docx]

**Supplementary Table S7** KEGG pathways of protein-coding transcripts with the highest density of line-specific missense variants identified in the Fat and Lean mouse selection lines.

| **Gene symbol** | **KEGG pathway** | **Line** |
| --- | --- | --- |
| *Ang5* | Amyotrophic lateral sclerosis | Lean |
| *Cd22* | Cell adhesion molecules, Hematopoietic cell lineage, B cell receptor signaling pathway | Fat |
| *Cx3cl1* | Viral protein interaction with cytokine and cytokine receptor, Efferocytosis, TNF signaling pathway, Chemokine signaling pathway, Cytokine-cytokine receptor interaction, Human cytomegalovirus infection | Fat |
| *H2-Aa* | Cell adhesion molecules, Hematopoietic cell lineage, Herpes simplex virus 1 infection, Phagosome, Inflammatory bowel disease, Type I diabetes mellitus, Leishmaniasis, Epstein-Barr virus infection, Systemic lupus erythematosus, Viral myocarditis, Graft-versus-host disease, Human T-cell leukemia virus 1 infection, Allograft rejection, Rheumatoid arthritis, Intestinal immune network for IgA production, Influenza A, Toxoplasmosis, Tuberculosis, Th1 and Th2 cell differentiation, Autoimmune thyroid disease, Staphylococcus aureus infection, Antigen processing and presentation, Asthma, Th17 cell differentiation | Lean |
| *H2-Ab1* | Cell adhesion molecules, Hematopoietic cell lineage, Herpes simplex virus 1 infection, Phagosome, Inflammatory bowel disease, Type I diabetes mellitus, Leishmaniasis, Epstein-Barr virus infection, Systemic lupus erythematosus, Viral myocarditis, Graft-versus-host disease, Human T-cell leukemia virus 1 infection, Allograft rejection, Rheumatoid arthritis, Intestinal immune network for IgA production, Influenza A, Toxoplasmosis, Tuberculosis, Th1 and Th2 cell differentiation, Autoimmune thyroid disease, Staphylococcus aureus infection, Antigen processing and presentation, Asthma, Th17 cell differentiation | Fat |
| *Hamp2* | TGF-beta signaling pathway | Fat, Lean |
| *Hbb-bh2* | Malaria, African trypanosomiasis | Lean |
| *Kcnmb2* | Vascular smooth muscle contraction, Insulin secretion, cGMP-PKG signaling pathway | Lean |
| *Nlrp1b* | NOD-like receptor signaling pathway | Fat |
| *Or8k28* | Olfactory transduction | Lean |
| *Or5w17* | Olfactory transduction | Lean |
| *Or10x4* | Olfactory transduction | Fat |
| *Or5p58* | Olfactory transduction | Fat |
| *Tas2r103* | Taste transduction | Fat |
| *Tas2r109* | Taste transduction | Fat |
| *Tas2r131* | Taste transduction | Fat |
| *Tas2r136* | Taste transduction | Fat |
| *Zfp426* | Herpes simplex virus 1 infection | Lean |
